# Disulfiram Alleviates Metabolic Dysfunction-Associated Steatohepatitis in Mice via Inhibiting Aurora Kinase A and Restoring Autophagy

**DOI:** 10.3390/antiox15070867

**Published:** 2026-07-11

**Authors:** Zixiong Zhou, Xi Zeng, Yuqi Guo, Zhengyi Tan, Xin Zhang, Xuyang Liu, Shuyu Zheng, Wenwen Liu, Haiyan Wang, Jing Qi

**Affiliations:** 1 Department of Pathology, Institute of Oncology, School of Basic Medical Sciences, Fujian Medical University, Fuzhou 350122, China; zzxpathology@fjmu.edu.cn (Z.Z.); 2240110094@fjmu.edu.cn (X.Z.); guoyuqi@stu.fjmu.edu.cn (Y.G.); tanzengyi@fjmu.edu.cn (Z.T.); 2250110079@fjmu.edu.cn (X.L.); zhengshuyu@stu.fjmu.edu.cn (S.Z.); wenzi0538@fjmu.edu.cn (W.L.); 2 Department of Biochemistry and Molecular Biology, School of Basic Medical Sciences, Fujian Medical University, Fuzhou 350122, China; 17860395950@fjmu.edu.cn

**Keywords:** MASH, disulfiram, AURKA, hepatocyte lipotoxicity, autophagy, drug repurposing

## Abstract

Metabolic dysfunction-associated steatohepatitis (MASH) is a severe, progressive liver disease lacking effective therapies. Disulfiram (DSF), an FDA-approved medication for alcohol dependence, exhibits diverse biological activities beyond its primary indication. This study aimed to evaluate whether DSF holds intervention promise for MASH and to unravel the underlying molecular mechanism. The efficacy of DSF was assessed in a mouse model of MASH induced by a choline-deficient, L-amino acid-defined diet, as well as in hepatocytes exposed to free fatty acids (FFAs) to trigger lipotoxicity. RNA-seq analysis combined with bioinformatic approaches was performed to identify key pathways and hub genes. Mechanistic validation was carried out using Western blotting and qPCR. Computational predictions suggested that DSF may influence insulin resistance, inflammation, autophagy-related markers, and lipid metabolism. In FFAs-treated hepatocytes, DSF administration dose-dependently reduced lipid accumulation and lipotoxicity. Consistently, in MASH mice, DSF administration significantly lowered elevated serum ALT (35%) and AST (40%) levels and the absolute hepatic triglyceride content (reduced from 1 to 0.5 μg/mg protein), and markedly attenuated hepatic steatosis, inflammation, fibrosis, and oxidative stress. Of note, RNA-seq analysis revealed that DSF modulated autophagy-related pathways and identified Aurora kinase A (AURKA) as a central downregulated hub gene. Mechanistically, DSF suppressed AURKA expression, which in turn led to changes in autophagy-related markers. These changes in autophagy-related markers were functionally coupled to a reduction in lipotoxicity. Collectively, DSF alleviates MASH by inhibiting AURKA, thereby relieving AURKA-mediated suppression of autophagy-related markers, which was associated with diminishing lipotoxicity, and ultimately achieving broad suppression of disease progression. Thus, DSF represents a promising hepatoprotective candidate for the intervention of MASH.

## 1. Introduction

Metabolic dysfunction-associated steatohepatitis (MASH) is the inflammatory and progressive subtype of metabolic dysfunction-associated steatotic liver disease (MASLD), the world’s most common chronic liver disease, and it is evolving into a severe public health crisis [1,2]. It is also important to acknowledge the evolving nomenclature in this field. The transition from non-alcoholic fatty liver disease (NAFLD) to metabolic dysfunction-associated fatty liver disease (MAFLD) and finally to the current MASLD reflects a deeper understanding of the disease’s pathophysiology and aims to reduce stigma, though this process has also generated considerable debate within the scientific community [3]. Its disease spectrum ranges from simple hepatic steatosis (MASL) to MASH, characterized by hepatocyte injury and inflammation, and can further progress to liver fibrosis, cirrhosis, and even hepatocellular carcinoma (HCC) [4]. Globally, approximately 30% to 40% of adults are affected by MASLD, with an estimated 3% to 6% having MASH. This prevalence is significantly higher among populations with obesity and type 2 diabetes and is projected to continue rising [1,2]. Notably, MASLD is not merely a liver-centric disease; it is a systemic metabolic disorder with significant extrahepatic manifestations, including an increased risk of cardiovascular disease, type 2 diabetes, chronic kidney disease, and various cancers, which further amplifies its global health burden [5].

While lifestyle interventions remain the cornerstone of management, long-term patient adherence is often suboptimal. Furthermore, patients with MASLD frequently experience a substantial decline in their health-related quality of life, reporting higher levels of fatigue, decreased physical functioning, and reduced work productivity, which underscores the importance of considering patient-reported outcomes in clinical management and intervention development [6]. The recent conditional approval of Resmetirom via the FDA Accelerated Approval pathway in March 2024 marked the first pharmacotherapy for MASH with fibrosis (F2–F3) [7,8]. While Phase 3 MAESTRO-MASH data showed histological improvement and lipid lowering, this approval remains conditional, requiring ongoing confirmation of clinical benefit [9]. Consequently, exploring novel intervention targets and strategies, particularly drug repurposing based on existing agents to accelerate clinical translation, represents an urgent priority in MASH research [10,11].

Autophagy, a lysosome-mediated intracellular degradation and recycling system, plays a central role in maintaining hepatic metabolic homeostasis [12]. It responds to metabolic stress by clearing damaged organelles, misfolded proteins, and, notably, lipid droplets [13,14]. Substantial evidence indicates that autophagic function is closely linked to the pathological progression of MASLD/MASH. Under predisposing conditions such as obesity and aging, hepatic autophagy levels are generally decreased. This functional impairment exacerbates intracellular lipid accumulation, insulin resistance, oxidative stress, and endoplasmic reticulum stress, thereby driving the transition from MASL to inflammatory hepatitis (MASH) [15,16]. For instance, studies have confirmed that in MASH models, autophagic flux exhibits dynamic changes, potentially compensatorily enhanced in early stages but often suppressed in later stages due to functional defects such as impaired fusion of autophagosomes with lysosomes [13]. Targeted enhancement of autophagy has been proven effective in alleviating hepatic steatosis, inflammation, and injury in preclinical models. Consequently, modulating autophagy has emerged as a highly promising direction for treating MASLD/MASH [14].

Aurora kinase A (AURKA) is a serine/threonine kinase traditionally of great interest due to its crucial role in regulating centrosome maturation, spindle assembly, and chromosome segregation during mitosis, with its overexpression associated with poor prognosis in various solid tumors [17,18]. However, increasing evidence in recent years has revealed that AURKA also plays important roles in non-mitotic cellular processes, particularly in regulating inflammation and autophagy [19]. For example, in non-small cell lung cancer, upregulated AURKA expression is linked to radioresistance, and its silencing can induce cytotoxic autophagy by downregulating the downstream factor CXCL5, thereby enhancing radiosensitivity [18]. Similarly, in a psoriasis model, AURKA exacerbates inflammatory responses by activating the AKT/mTOR pathway and blocking autophagy-mediated suppression of the AIM2 inflammasome [19]. These findings suggest that AURKA may be a critical node connecting cellular stress, autophagy, and inflammatory responses. However, its regulatory role in autophagy and the underlying mechanisms in MASH, a metabolic inflammatory disease, remain unclear, leaving room for exploring new intervention targets.

Disulfiram (DSF) is a drug that has been approved by the FDA for decades for treating chronic alcohol dependence, with a relatively well-established safety record [20]. In recent years, drug repurposing research has discovered that DSF and its active metabolites exhibit broad biological activities far beyond their original indication, including significant anti-tumor, anti-viral, and anti-parasitic benefits, as well as potential benefits in improving metabolic diseases [21,22,23]. Its mechanisms of action are complex and pleiotropic, involving the regulation of multiple molecular targets. One of the most well-known mechanisms is that diethyldithiocarbamate (DDC), formed from DSF metabolism in vivo, can complex with copper ions to form CuET. This complex can target and inhibit NPL4, an adaptor protein for the p97/VCP segregase, thereby disrupting cellular protein homeostasis, inducing the unfolded protein response, and ultimately leading to cancer cell death [24]. Furthermore, DSF/DDC has been shown to directly inhibit the chemokine signal regulator FROUNT in tumor-associated macrophages, reducing macrophage recruitment into tumors and exerting anti-tumor immunomodulatory effects [21]. Notably, its derivative DDC also demonstrates multiple benefits in alleviating oxidative stress, ER stress, and inflammation [25,26]. However, the specific molecular mechanisms underlying these metabolic protective effects of DSF currently remain unknown.

Based on the above background, we hypothesized that DSF might alleviate the pathogenesis of MASH. Accordingly, the main objectives of this study were to elucidate whether DSF is intervenable for MASH and to illustrate the underlying mechanism by which DSF blocks disease progression.

## 2. Materials and Methods

### 2.1. Chemicals and Reagents

**Figure 1:**
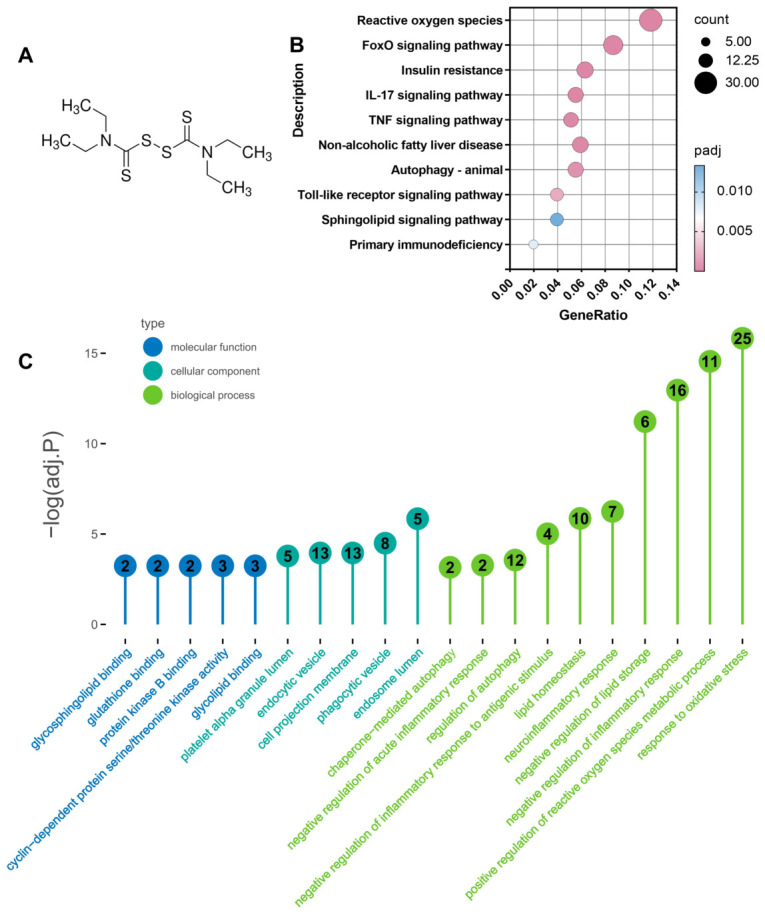
Molecular structure of DSF and its predicted biological functions through target enrichment analysis. (**A**) Two-dimensional molecular structure of DSF. (**B**) KEGG pathway enrichment analysis for potential targets of DSF predicted by PharmMapper. (**C**) GO enrichment analysis for DSF-predicted targets.

DSF (CAS # 97-77-8) was acquired from a biotechnology company called Haoyuan Biotechnology Co., Ltd., located in Shanghai, China. Moreover, after the determination and analysis of DSF by HPLC, the data showed that the purity of DSF was more than 99%. Figure 1A shows the two-dimensional molecular structure of DSF. The AURKA activator (anacardic acid, CAS # 16611-84-0), with a purity of ≥98%, as determined by HPLC analysis, was acquired from Aladdin Biochemical Technology Co., Ltd., located in Shanghai, China.

### 2.2. Animal

This investigation employed eight-week-old male C57BL/6N mice. All breeding and housing procedures were performed under standard specific pathogen-free conditions, maintained at a temperature of 25 ± 1 °C and 50 ± 5% relative humidity, with a 12 h light/dark cycle. Throughout the study, animals had ad libitum access to autoclaved water and feed. The experimental protocol was approved by the Laboratory Animal Research Center of Fujian Medical University (ethical code: FJMU IACUC 2024-Y-2238, 8 October 2024) and complied with the guidelines authorized by the National Association of Laboratory Animal Care, ensuring all procedures met ethical standards.

To assess the administration efficacy of DSF against MASH induced by a choline-deficient, L-amino acid-defined (CDAA) diet, fifteen mice were randomly allocated into three experimental groups (*n* = 5 per group): a normal diet (ND)-treated group, a MASH model group, and a MASH model + DSF administration group. The MASH model was established via continuous feeding with the CDAA diet. Mice in the administration group subsequently received intraperitoneal injections of DSF (25 mg/kg), administered three times per week for four weeks. The injection volume was adjusted according to individual body weight. The ND group received isovolumetric intraperitoneal injections of saline on an identical schedule. Following the four-week administration period, all mice were euthanized, and relevant biological specimens were collected for subsequent analysis.

### 2.3. Potential Target Protein Identification for DSF

The canonical two-dimensional chemical structure of DSF was retrieved from the PubChem database using its corresponding CAS registry number. Subsequently, the PharmMapper server was employed to predict potential target proteins for DSF through reverse pharmacophore matching. The resulting list of target proteins was standardized by mapping the protein identifiers to official gene symbols using the UniProt database, followed by the removal of duplicate entries. Finally, Gene Ontology (GO) functional annotation and Kyoto Encyclopedia of Genes and Genomes (KEGG) pathway enrichment analyses were performed on the curated gene list using the DAVID bioinformatics resource (https://david.ncifcrf.gov/, accessed on 13 May 2025).

### 2.4. RNA-Sequencing Analysis

Total RNA was isolated from tissue specimens using Trizol reagent (Invitrogen, Carlsbad, CA, USA), followed by removal of genomic DNA via DNase I treatment (TaKaRa, Dalian, China). The integrity and purity of the RNA were assessed using an Agilent 2100 Bioanalyzer (Agilent, Santa Clara, CA, USA), and concentration was measured with a NanoDrop ND-2000 spectrophotometer (NanoDrop Technologies, Wilmington, DE, USA). Sequencing libraries for transcriptome analysis were constructed from 1 µg of total RNA using the TruSeq RNA Sample Preparation Kit (Illumina, San Diego, CA, USA). Paired-end sequencing (150 bp × 2) was performed on an Illumina NovaSeq 6000 platform at Shanghai BIOZERON Co., Ltd., Shanghai, China. Raw paired-end reads were preprocessed with Trimmomatic (version 0.36) under the following parameters: SLIDINGWINDOW:4:15 and MINLEN:75. High-quality clean reads were then aligned to the reference genome using HISAT2 with default settings. Post-alignment quality control was executed using Qualimap (version 2.2.1), and gene-level read counts were quantified with HTSeq (release 0.11.1). Gene expression levels were determined using the FPKM method. For differential expression analysis, the R package edgeR (version 4.5.1) was employed, and differentially expressed genes (DEGs) were identified based on thresholds of |log2 fold change| > 2 and an FDR < 0.05. Functional enrichment analysis, including Gene Ontology (GO) and Kyoto Encyclopedia of Genes and Genomes (KEGG) pathway analyses, was conducted using Goatools (version 1.0.2) and KOBAS (version 3.0, http://kobas.cbi.pku.edu.cn/, accessed on 16 March 2025), respectively, with statistical significance set at a Bonferroni-corrected *p*-value < 0.05.

### 2.5. Biochemical Assays

Cardiac blood samples were collected from mice. The activities of alanine aminotransferase (ALT) and aspartate aminotransferase (AST) in the serum were determined using commercial assay kits (Nanjing Jiancheng Bioengineering Institute, Nanjing, China; Jianglai Bio, Shanghai, China). Liver tissues were homogenized in PBS by ultrasonication and centrifuged at 10,000 *g* for 10 min to obtain the supernatant. Subsequently, the levels of total cholesterol (TC) and triglycerides (TG) in the supernatant were measured using corresponding commercial kits (Nanjing Jiancheng Bioengineering Institute, Nanjing, China; Jianglai Bio, Shanghai, China) to assess hepatic lipid accumulation.

### 2.6. Staining of Liver Sections

As described before [27], liver tissues were fixed overnight, embedded in paraffin, and sectioned. Sections were stained using a Hematoxylin and Eosin (H&E) staining kit (G1125, Solarbio, Beijing, China) or by the terminal deoxynucleotidyl transferase dUTP nick end labeling (TUNEL) method (In Situ Cell Death Detection Kit, POD, 11684817910, Roche, Basel, Switzerland) according to the manufacturers’ protocols. For immunohistochemistry (IHC), sections were incubated overnight at 4 °C with primary antibodies against myeloperoxidase (MPO, 1:500, 66177-1-Ig, Proteintech, Wuhan, China), F4/80 (1:200, sc-377009, Santa Cruz, Dallas, TX, USA), CD86 (1:300, sc-28347, Santa Cruz), and CD163 (1:2000, 16646-1-AP, Proteintech, Wuhan, China). Subsequently, sections were treated with appropriate secondary antibodies and counterstained with hematoxylin. The proportion of positive areas was semi-quantitatively analyzed using ImageJ (version 1.52) software. Furthermore, H&E-stained sections were evaluated by two professional pathologists according to the steatosis grade, lobular inflammation, and MASLD activity score (MAS). Additionally, to assess hepatic lipid content, thick sections were stained with Oil Red O, and Sirius-red dye was used to detect hepatic fibrosis accumulation.

### 2.7. Quantitative Real-Time Polymerase Chain Reaction (qRT-PCR)

As detailed in reference [28], Total RNA was extracted from liver tissues and cells using TRIzol reagent, followed by RNA concentration measurement and equal-concentration dilution. RNA was reverse transcribed into cDNA using the HRbioTMIII First Strand cDNA Synthesis Kit (OneStep gDNA removal method) (Fujian Herui Biotechnology Co., Ltd., Fuzhou, China). A qRT-PCR system was constructed using PCR premix (E00019, GenScript Biotech Corporation, Nanjing, China) to evaluate the transcriptional levels of relevant genes. The primer sequences used in this study are listed in Table 1. Relative gene expression was calculated using the 2^−ΔΔCt^ method, where the target gene Ct values were first normalized to the internal reference gene (GAPDH) to obtain ΔCt values, and then the fold change relative to the control group was calculated as 2^−ΔΔCt^.

### 2.8. Western Blot Analysis

The procedure followed reference [29], Protein samples were extracted from liver tissues on ice using RIPA lysis buffer (Beyotime Biotechnology, Shanghai, China). After centrifugation (12,000 *g*, 15 min, 4 °C), the supernatant was collected, and protein concentration was determined using a BCA assay kit (Beyotime Biotechnology). All samples were diluted to a uniform concentration of 3.75 mg/mL. Following denaturation by boiling for 5 min, proteins were separated by 10% SDS-PAGE and transferred onto a polyvinylidene difluoride membrane (IPVH00010, Millipore, Billerica, MA, USA) in an ice-water bath. The membrane was blocked with 5% skim milk at room temperature for 1 h and then incubated overnight at 4 °C with primary antibodies against Aurka (11531-1-AP, Proteintech, Wuhan, China), Tim23 (#2772S), Parkin (#3498S), TOMM40 (19677-1-AP, Proteintech), LC-3B (#2922S), and β-actin (abs171598, Absin, Shanghai, China). After washing three times with TBST, the membrane was incubated with a horseradish peroxidase-conjugated secondary antibody (SA00001-7L, Proteintech) at room temperature for 1 h. Immunoreactive bands were visualized using an ultra-sensitive ECL kit (Fujian Herui Biotechnology Co., Ltd., Fuzhou, China), and images were captured with an electrophoresis gel imaging system (Peiqing Technology, Shanghai, China). Relative protein expression levels were quantified using ImageJ software.

### 2.9. Cell Culture

AML12 cells (ATCC CRL-2254, immortalized mouse hepatocytes) obtained from the American Type Culture Collection., Ltd. (Manassas, VA, USA), were cultured in DMEM/F-12 medium supplemented with 10% fetal bovine serum, 40 ng/mL dexamethasone, antibiotics, and an insulin–transferrin–selenium mixture, and maintained in a humidified incubator at 37 °C with 5% CO_2_. To evaluate the protective effect of DSF against lipotoxicity, hepatocytes were treated with PO (0.4 mM palmitic acid: 0.8 mM oleinic acid) in the presence or absence of DSF (25, 50, and 100 μM) for 24 h, after which cell samples were collected for subsequent analysis. Neutral lipid droplets in hepatocytes were detected using Bodipy staining (MedChemExpress, Shanghai, China). Briefly, cells were rinsed with cold PBS to remove the culture medium, incubated with 2 μM Bodipy staining solution for 15 min, and then observed under a fluorescence microscope (IX70, Olympus, Tokyo, Japan) to capture images of green-stained lipid droplets. Additionally, an AURKA activator (Anacardic acid, 60 μM) was used to confirm whether AURKA is a necessary target for the protective effects provided by DSF in vitro.

### 2.10. Cell Viability Assay

Cells were seeded into culture plates, and the viability of hepatocytes under lipotoxic conditions was assessed using the Cell Counting Kit-8 (CCK-8) assay. The absorbance of different samples was measured using an EMax spectrophotometer (Invitrogen, Carlsbad, CA, USA).

### 2.11. Statistical Analysis

Statistical analysis was performed using GraphPad Prism software (version 9.0, GraphPad Software, San Diego, CA, USA). All data are presented as mean ± standard deviation (SD). Comparisons between two groups were analyzed using a two-tailed Student’s *t*-test, with asterisks indicating statistically significant differences. Comparisons among multiple groups were performed using one-way analysis of variance (ANOVA) followed by the Tukey–Kramer post hoc test (*p* < 0.05). Means labeled with the same letter are not statistically different, whereas those labeled with different letters are statistically significant. All parametric tests were Kruskal–Wallis tests, and Dunn’s post hoc test was used for multiple comparisons. The histopathological scoring of liver tissue sections was conducted using ImageJ software (version 1.8.0.112).

## 3. Results

### 3.1. Computational Predictions of DSF Functional Pathways

Firstly, the PharmMapper server was used to predict 290 potential DSF target proteins (Figure 1A), and these proteins were subsequently submitted to the DAVID database (v6.8, https://david.ncifcrf.gov/, accessed on 13 May 2025.) for GO and KEGG enrichment analysis. A total of 2056 GO terms with *p*-values less than 0.05 and 163 KEGG terms were generated. KEGG enrichment terms include non-alcoholic fatty liver disease and autophagy (Figure 1B). Enriched GO terms for CC (*n* = 31), BP (*n* = 1756), and MF (*n* = 268) were generated, which mainly include negative regulation of inflammatory response, negative regulation of lipid storage, regulation of lipid transport, regulation of autophagy, and oxidative stress (Figure 1C). These analyses suggest that DSF might have a retarding effect on the progression of MASH.

### 3.2. DSF Reduces Lipid Accumulation in Hepatocytes Under Metabolic Stress

**Figure 2:**
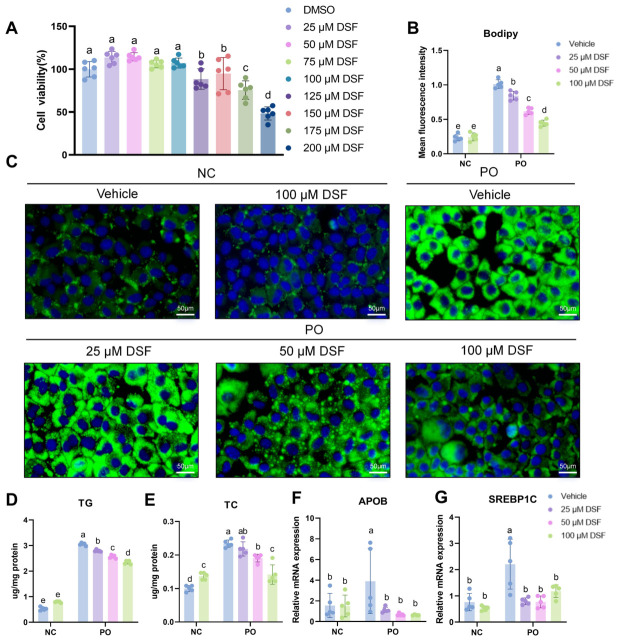
DSF reduces lipid accumulation in hepatocytes under metabolic stress. (**A**) Cell viability of hepatocytes treated with different concentrations of DSF was detected by CCK8. Concentrations above 100 µM were excluded from functional assays due to the dose-dependent drop in cell viability seen in panel (**B**) Quantitative analysis of Bodipy mean fluorescence intensity. (**C**) Representative fluorescence microscopy images of Bodipy staining showing lipid droplets in hepatocytes (×400) (bar, 50 μm). Bodipy fluorescence (green) represents lipid deposition. (**D**,**E**) Triglycerides (TG) and total cholesterol (TC) were measured. (**F**,**G**) Relative mRNA expression levels of key genes (APOB and SREBP1c) involved in lipid metabolism or transport in hepatocytes were assessed by qRT-PCR. Data are expressed as mean ± SD in each group; statistical significance was determined by one-way ANOVA among several variables at *p* < 0.05, which are represented by different letters. All parametric tests were Kruskal–Wallis tests, and Dunn’s post hoc test was used for multiple comparisons.

To further confirm whether DSF can directly diminish lipid accumulation in hepatocytes, a fatty liver toxicity model in AML12 cells was constructed using PO. As shown in Figure 2A, DSF administration at concentrations up to 100 μM for 24 h did not induce significant cytotoxicity compared to the vehicle control. Based on these results, we selected three non-toxic concentrations (25, 50, and 100 μM) for subsequent mechanistic studies to ensure that observed effects were attributable to pharmacological modulation rather than general cytotoxicity. We also used Bodipy fluorescent dye to demonstrate the intracellular lipid content and found that treating DSF attenuated the excessive lipid deposition in lipotoxic hepatocytes in a dose-dependent manner (Figure 2B,C). Moreover, DSF dose-dependently decreased TG and TC levels in PO-treated hepatocytes (Figure 2D,E). Notably, the mRNA expression levels of relevant lipid metabolism genes, namely SREBP-1c and APOB, also exhibited a similar pattern (Figure 2F,G). Accordingly, DSF could reduce lipid accumulation in lipotoxic hepatocytes.

### 3.3. DSF Alleviates Disease Progression in a Mouse MASH Model

**Figure 3:**
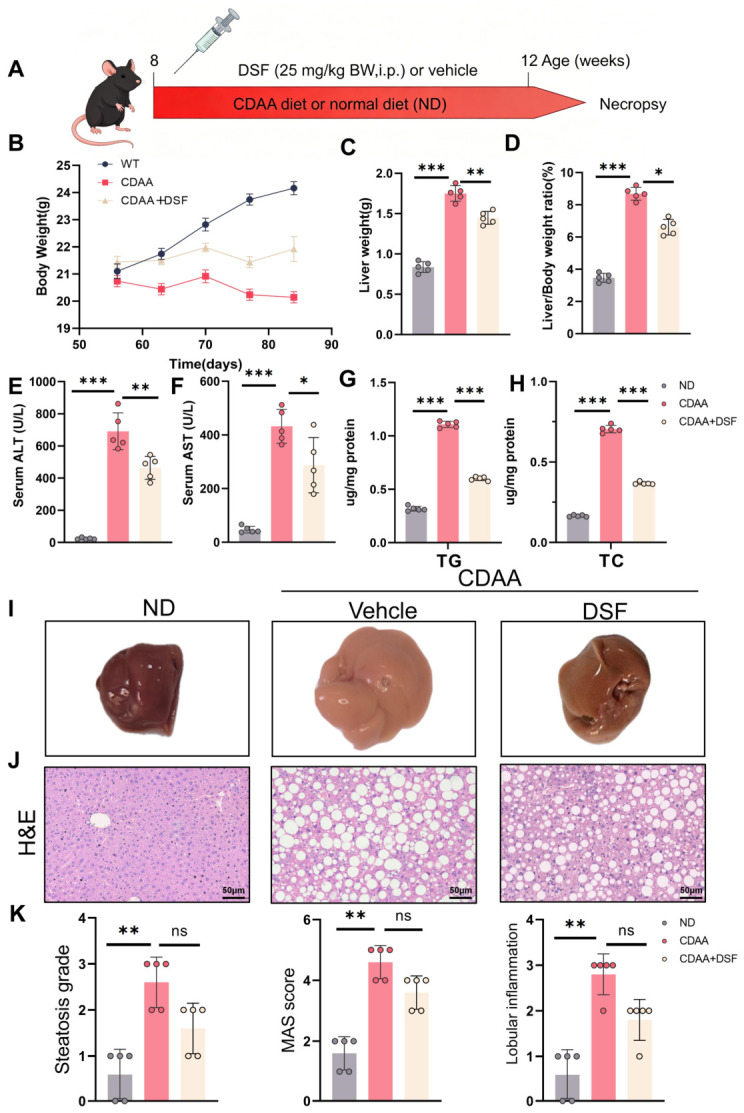
DSF ameliorates metabolic and histological parameters in a CDAA diet-induced murine MASH model. (**A**) Mice were given a CDAA diet for a duration of 4 weeks. Animals in each group received intraperitoneal injections of DSF (25 mg/kg) or an equivalent volume of vehicle three times per week. Body weight (**B**), liver weight (**C**), and liver-to-body weight ratio (**D**) were recorded. (**E**) Serum ALT levels. (**F**) Serum AST levels. (**G**) Hepatic TG content. (**H**) Hepatic TC content. (**I**) Representative photographs of livers from each group. (**J**,**K**) Representative H&E staining of liver sections from each group, sowing steatosis and ballooning, nuclei are stained blue/purple, cytoplasm is pink/red, and the white circular areas represent lipid droplets (steatosis). (×400) (bar, 50 μm). Data are presented as mean ± SD. The Kruskal–Wallis test with Dunn’s post hoc test was used to analyze the histological scores (steatosis grade, MAS score and lobular inflammation). * *p* < 0.05, ** *p* < 0.01, *** *p* < 0.001, ns (not significant) vs. indicated groups.

Next, we conducted animal experiments to further validate the effect of DSF on hepatocyte lipid deposition in MASH. Mice were grouped and treated according to the regimen outlined in Figure 3A. The MASH model was induced using a CDAA diet, with concurrent DSF (25 mg/kg) administration, without significant hepatotoxicity (Figure S1). Following a 4-week feeding period, samples were collected. The CDAA diet successfully established the murine MASH model, as confirmed by reduced body weight; increased liver weight, liver-to-body weight ratio, and serum ALT and AST levels; and elevated hepatic TG and TC levels. However, DSF intervention reversed these changes (Figure 3B–H), consistent with expectations. Furthermore, liver tissues from model mice exhibited varying degrees of hepatomegaly, yellowish discoloration, increased fragility, and pronounced lipid droplet accumulation (Figure 3I–K). Importantly, these pathological alterations were markedly reversed upon DSF administration. Thus, DSF could attenuate the progression of MASH in mice.

### 3.4. Bioinformatics Analysis of RNA-Seq

**Figure 4:**
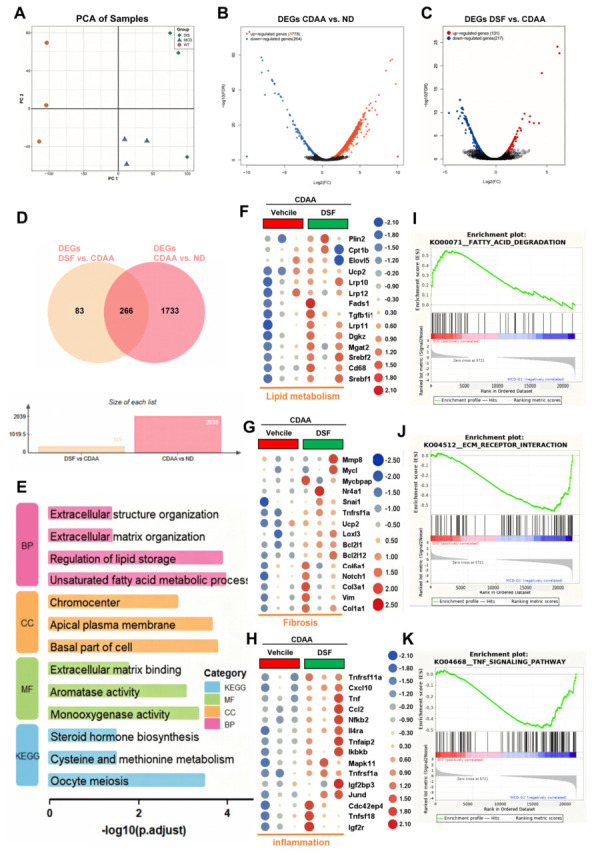
Transcriptomic analysis reveals that DSF modulates pathways related to lipid metabolism, fibrosis, and inflammation in MASH livers. (**A**) PCA plot of liver transcriptomes from each group. (**B**) Volcano plot of DEGs between CDAA-treated and ND-treated groups. (**C**) Volcano plot of DEGs between CDAA + DSF and CDAA groups. In the volcano plots, red points represent significantly upregulated DEGs, blue points represent significantly downregulated DEGs, and grey points represent non-significant genes, based on fold change > 1.5 and adjusted *p* < 0.05. (**D**) Venn diagram showing the intersection of DEGs from different comparison groups. (**E**) GO and KEGG functional enrichment analysis. (**F**–**K**) Heatmaps of DEGs and GSEA plots for specific pathways indicated that DSF ameliorated MASH-related liver complications, including decreased lipid deposition, fibrosis, and inflammation. In the GSEA plots, the red and blue curves indicate gene sets positively and negatively correlated with the phenotype, respectively. The green line represents the running enrichment score, and the bottom color bar reflects the ranked gene list.

To systematically elucidate the mechanism by which DSF prevents MASH progression, we performed RNA-seq on liver tissue samples. Principal component analysis (PCA) revealed clear separation among the experimental groups (Figure 4A). Analysis of DEGs showed that, compared with the ND-treated group, the CDAA diet-treated group exhibited 1773 significantly upregulated genes and 264 downregulated genes (Figure 4B). In contrast, DSF administration in the CDAA-fed group resulted in 131 upregulated and 217 downregulated genes relative to the CDAA group alone (Figure 4C). Functional enrichment analysis was performed on the 266 overlapping DEGs identified from these comparisons (Figure 4D), using the KEGG and GO databases. The results demonstrated that these DEGs were significantly enriched in pathways associated with lipid metabolism and biological processes related to extracellular matrix organization and fibrosis (Figure 4E). Furthermore, heatmap analysis of DEGs and GSEA results showed the efficacy of DSF in alleviating hepatic fat accumulation, fibrosis, and inflammation in the MASH model (Figure 4F–K). Collectively, these analyses further indicated that DSF modulates lipid metabolism in MASH mice.

### 3.5. DSF Intervention Alleviates Hepatic Steatosis, Fibrosis, and Inflammation in MASH Mice

**Figure 5:**
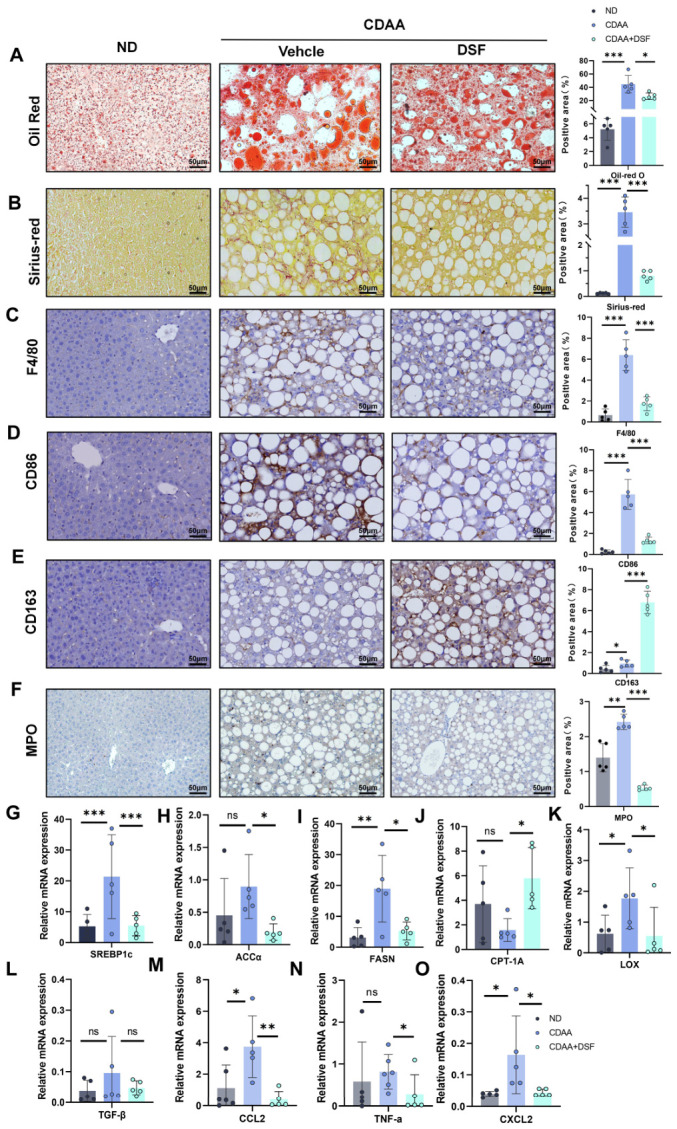
DSF ameliorates hepatic steatosis, fibrosis, and inflammation in MASH mice. (**A**) Representative images and quantitative analysis of Oil Red O staining for hepatic lipid droplets, lipid droplets are stained red in Oil Red O staining. (**B**) Representative images and quantitative analysis of Sirius-red staining for fibrosis, collagen fibers are stained red in Sirius-red staining. (**C**–**F**) Representative IHC staining and quantitative analysis of immune cell infiltration: (**C**) F4/80+ total macrophages, (**D**) CD86^+^ M1 macrophages, (**E**) CD163^+^ M2 macrophages, and (**F**) MPO^+^ neutrophils (×400) (bar, 50 μm). (**G**–**O**) qRT-PCR analysis of mRNA expression for genes involved in (**G**–**I**) lipogenesis (SREBP1c, ACCα, FASN), (**J**) fatty acid oxidation (CPT-1A), (**K**,**L**) fibrosis (LOX, TGF-β), and (**M**–**O**) inflammation (CCL2, TNF-α, and CXCL2). Data are presented as mean ± SD. * *p* < 0.05, ** *p* < 0.01, *** *p* < 0.001, ns (not significant) vs. indicated groups.

Given that dysregulated lipid metabolism, inflammation, and fibrosis are core pathological features driving the progression of MASH towards end-stage liver disease [30,31], we systematically evaluated the effects of DSF on these aspects (Figure 5A–O). Hepatic lipid metabolic imbalance is the initiating event and central characteristic of MASH development, and the resulting lipotoxicity is a key factor driving hepatocyte injury and inflammation [32]. Hepatic fibrosis is the primary pathological basis for the progression of MASH to cirrhosis and liver failure, and is a critical determinant of patient prognosis [33].

Oil Red O staining results showed that, compared to the ND group, the livers from the MASH model exhibited numerous lipid droplets, indicating severe steatosis. DSF intervention significantly reduced the area and number of lipid droplets in liver sections, demonstrating its efficacy in alleviating hepatic fat accumulation (Figure 5A). Consistently, CDAA diet upregulated lipogenesis-related genes and downregulated fatty acid β-oxidation-related genes, whereas DSF reversed these effects (Figure 5G–J). Sirius-red staining results showed that the CDAA diet induced marked hepatic fibrosis, which was attenuated by DSF intervention (Figure 5B). At the molecular level, DSF reduced the expression of pro-fibrotic genes, including LOX and TGF-β (Figure 5K–L). IHC analysis demonstrated that DSF suppressed the infiltration of F4/80^+^ and CD86^+^ macrophages and neutrophils (MPO^+^), while increasing the infiltration of CD163 macrophages in MASH mouse livers (Figure 5C–F). These findings were confirmed at the transcriptional level: DSF downregulated mRNA levels of inflammatory mediators such as TNF-α, CCL2, and CXCL2 (Figure 5M–O). Jointly, these findings demonstrate that DSF optimizes hepatic lipid metabolism and exerts significant anti-fibrotic and anti-inflammatory properties, thereby mitigating the pathological progression and overall severity of MASH.

### 3.6. DSF Alleviates Hepatocyte Injury in MASH by Restoring Autophagy via AURKA Inhibition

**Figure 6:**
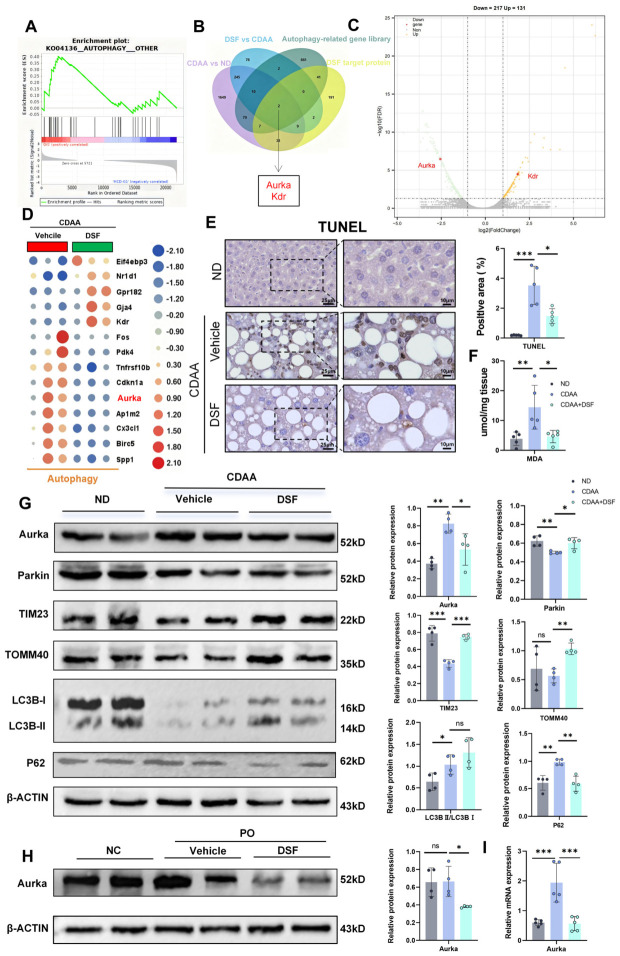
DSF alleviates MASH by downregulating AURKA to restore impaired hepatic autophagy. (**A**) GSEA shows that DSF intervention significantly enriches pathways related to autophagy. In the GSEA plots, the red and blue curves indicate gene sets positively and negatively correlated with the phenotype, respectively. The green line represents the running enrichment score, and the bottom color bar reflects the ranked gene list (**B**) Integration of differential gene expression profiles, target prediction, and autophagy gene databases. (**C**) Volcano plot of DEGs. (**D**) Heatmap focusing on AURKA and its associated gene network. (**E**) Representative images of TUNEL staining (up: ×800, bar: 25 μm; down: ×1600, bar: 10 μm). (**F**) Hepatic MDA content. (**G**) Western blot was used to detect the expression levels of AURKA, Parkin, TIM23, TOMM40, LC3B-I, LC3B-II, P62, and β-actin in mouse liver tissues. The β-actin loading control was derived from the same membrane or from a parallel gel. (**H**) Western blot was used to detect the expression levels of AURKA and β-actin in AML12 cells. The β-actin loading control was derived from the same membrane or from a parallel gel. (**I**) qRT-PCR analysis showed the mRNA expression levels of AURKA in vitro. Data are presented as mean ± SD. * *p* < 0.05, ** *p* < 0.01, *** *p* < 0.001, ns (not significant) vs. indicated groups.

Then, we performed transcriptomic analysis to elucidate the molecular mechanism by which DSF ameliorates MASH. GSEA analysis revealed significant modulation of autophagy-related pathways by DSF (Figure 6A), suggesting that changes in autophagy-related markers are a key component of DSF’s intervention. By integrating DEGs, drug target prediction, and autophagy databases, we identified AURKA as a core hub gene downregulated by DSF (Figure 6B). Volcano plot and heatmap analyses confirmed AURKA as one of the most significantly reduced genes following DSF administration (Figure 6C,D).

Given the central role of autophagy in hepatic lipid metabolism and MASH progression [34,35] and AURKA in autophagy inhibition [19], we hypothesized that DSF alleviates MASH in association with changes in autophagy-related markers and AURKA, thereby reducing hepatocyte lipotoxicity and blunting MASH pathogenesis. DSF markedly reduced the increased apoptotic cell count and oxidative stress in MASH livers (Figure 6E,F). In vivo, Western blot analysis indicated that MASH livers had elevated AURKA protein expression alongside altered autophagy-related markers, as evidenced by decreased conversion of LC3B-I to LC3B-II, accumulation of P62, and reduced expression of the mitophagy markers (Parkin, TIM23, and TOMM40). DSF effectively downregulated AURKA protein levels and concurrently reversed these molecular changes (increased LC3B-II, Parkin, TIM23, and TOMM40, decreased P62) (Figure 6G). qPCR confirmed DSF significantly downregulated AURKA mRNA expression (Figure S2), aligning with the protein data. We further validated this regulatory axis in vitro using PO-treated hepatocytes to mimic lipotoxic stress. Western blot analysis showed that DSF similarly reduced AURKA protein expression in the cells (Figure 6H). qPCR results showed a similar pattern (Figure 6I).

Collectively, these data demonstrate that DSF downregulates AURKA both in vivo and in vitro, accompanied by alterations in autophagy-related markers. These changes were associated with clearance of lipid droplets and damaged organelles, reduces lipotoxicity, and attenuates hepatocyte apoptosis, thereby ameliorating MASH pathology.

### 3.7. AURKA Activation Abolishes DSF’s Effects on Lipotoxicity and Autophagy

**Figure 7:**
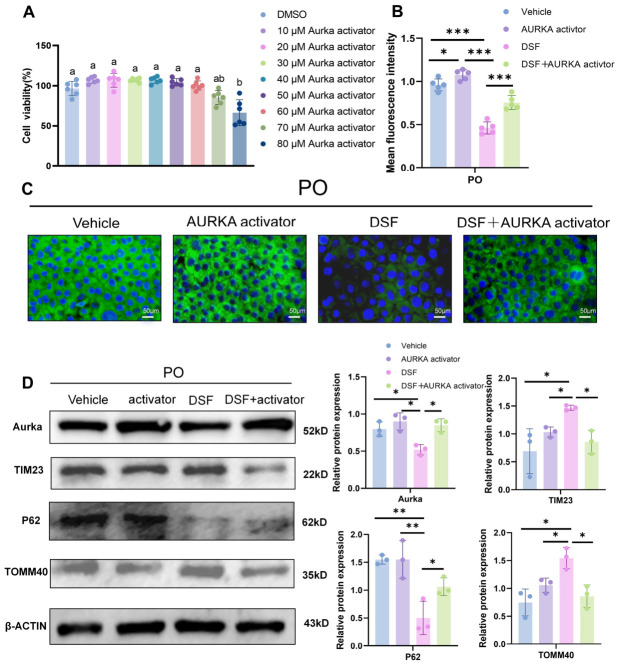
AURKA activation abolishes DSF’s effects on lipotoxicity and autophagy. (**A**) Cell viability of hepatocytes treated with different concentrations of AURKA activator was detected by CCK8. Data are expressed as mean ± SD in each group; statistical significance was determined by one-way ANOVA among several variables at *p* < 0.05, which are represented by different letters. (**B**) Quantitative analysis of Bodipy mean fluorescence intensity. (**C**) Representative fluorescence microscopy images of Bodipy staining showing lipid droplets in hepatocytes (×400) (bar, 50 μm). Bodipy fluorescence (green) represents lipid deposition. (**D**) Western blot was used to detect the expression levels of AURKA, TIM23, P62, TOMM40, and β-actin in AML12 cells. The β-actin loading control was derived from the same membrane or from a parallel gel. Data are presented as mean ± SD. * *p* < 0.05, ** *p* < 0.01, *** *p* < 0.001 vs. indicated groups. All parametric tests were Kruskal–Wallis tests, and Dunn’s post hoc test was used for multiple comparisons.

To confirm whether AURKA is a necessary target for the protective effects mediated by DSF and to verify whether changes in autophagy-related markers truly reflect changes in autophagy-related markers, we further employed a specific AURKA activator in pharmacological intervention experiments. Given that concentrations below 60 μM had no significant cytotoxicity to AML12 cells (Figure 7A), this concentration was selected for subsequent mechanistic validation. The results showed that in a lipotoxic environment induced by PO, the AURKA activator significantly reversed the mitigating effect of DSF on hepatocyte lipid deposition, manifested as increased BODIPY fluorescence intensity and lipid droplet area (Figure 7B,C). Concurrently, the alterations of key autophagy-related markers by DSF was also attenuated by the AURKA activator, accompanied by the re-accumulation of the autophagic substrate p62 and decreased levels of TIM23 and TOMM40 (Figure 7D). These results suggest that activating AURKA significantly blocks the ameliorative effects of DSF on hepatocyte lipotoxicity in vitro, thereby providing the first direct evidence that inhibition of AURKA is associated with the hepatoprotective effects of DSF.

## 4. Discussion

This study proposes and validates a novel strategy to repurpose DSF, an FDA-approved drug for alcohol dependence, as an intervention agent for MASH. Our findings demonstrate that DSF significantly ameliorated key MASH-associated complications in mice, including hepatocyte lipotoxicity, liver fibrosis, inflammation, and oxidative stress (Figure 3, Figure 4 and Figure 5). In vitro experiments further confirmed that DSF reduced FFA-induced lipid deposition in hepatocytes (Figure 2B–G). Collectively, these results indicate that DSF effectively mitigates core pathological phenotypes of MASH both in vivo and in vitro. Notably, given its decades-long clinical use, DSF has a well-characterized human safety profile, which offers a distinct advantage for rapid clinical translation [21]. In recent years, the pleiotropic pharmacological activities of DSF, attributed primarily to its metabolite-mediated targeting of zinc-finger-containing proteins such as the p97/NPL4 complex, have led to the discovery of its “new uses” in oncology, antiviral, and antiparasitic therapies [23]. This present study adds a new dimension to DSF’s pharmacological profile: intervention in metabolic diseases.

It should be noted that DSF is extensively metabolized in the liver and has been associated with rare but serious hepatotoxicity in clinical use. Since MASH patients often have pre-existing hepatic impairment, they may be particularly susceptible to such adverse effects. Therefore, the safety conclusions from this study remain preliminary, and dedicated dose-escalation and safety studies in MASH/MASLD models are warranted before clinical translation.

Autophagy, a lysosome-mediated intracellular degradation and recycling system, plays a central role in maintaining hepatocyte metabolic homeostasis and exerts a crucial protective function in MAFLD/MASH by degrading lipid droplets (lipophagy) and damaged organelles such as mitochondria [34,35]. Hepatic autophagic function is intricately intertwined with lipid metabolism. Fundamental research indicates that autophagy is a vital pathway for regulating hepatocyte lipid homeostasis. Through lipophagy, it directly degrades lipid droplets, releasing FFAs for mitochondrial β-oxidation, thereby reducing intrahepatic lipid accumulation [11]. Under metabolic stress conditions such as obesity and aging, hepatic autophagy is generally suppressed. This functional impairment not only reduces lipid droplet clearance but also diminishes fatty acid oxidation for energy, thereby exacerbating hepatocyte steatosis, insulin resistance, and oxidative stress, driving disease progression from simple steatosis to MASH [16]. In this study, the MASH model mouse livers exhibited marked alterations in autophagy-related markers, evidenced by reduced levels of the autophagosome marker LC3B-II, while P62 expression increased, and expression of the mitophagy marker Parkin decreased alongside mitochondrial proteins (TIM23, TOMM40), indicating alterations consistent with reduced autophagy-related markers, including mitophagy-related markers. Treating DSF effectively reversed these molecular changes, increasing LC3B-II levels, reducing P62 expression, and elevating the expression of Parkin, TIM23, and TOMM40 (Figure 6G and Figure S2). Ultimately, these changes in autophagy-related markers were closely associated with a significant reduction in hepatocyte apoptosis (Figure 6E). Autophagy is closely related to oxidative stress, as autophagy is known to helps to remove oxidative damage to cellular components, thereby maintaining cell homeostasis (Figure 6F). These findings directly link the metabolic protective effects of DSF to changes in autophagy-related markers.

Mechanistically, this study links the efficacy of DSF to the AURKA–autophagy regulatory axis. Transcriptomic analysis revealed that DSF intervention significantly enriched autophagy-related pathways (Figure 6A) and identified AURKA as a pivotal hub gene (Figure 6B–D). Experimental validation demonstrated that DSF significantly downregulated the aberrantly elevated expression of AURKA at both mRNA and protein levels in MASH livers (Figure 6G–I). AURKA, a serine/threonine kinase classically involved in mitotic regulation, is overexpressed in various cancers and promotes tumor progression [36,37]. However, accumulating evidence has proven that AURKA regulates non-mitotic processes, including autophagy inhibition. For instance, in psoriasis, AURKA exacerbates inflammation by blocking autophagy-mediated suppression of the AIM2 inflammasome [19]; in non-small cell lung cancer, inhibition of the AURKA-CXCL5 axis induces autophagic cell death [18]. Our study extends this understanding to metabolic liver disease, revealing that AURKA expression is upregulated in MASH and exacerbates disease progression by association with reduced autophagy-related markers. Therefore, the core mechanistic pathway elucidated is: DSF alleviates MASH pathogenesis in association with changes in autophagy-related markers and AURKA downregulation, which contributes to the relief of hepatocyte lipotoxicity.

To functionally validate the role of AURKA in DSF’s hepatoprotective mechanism, we performed gain-of-function experiments using a specific pharmacological activator of AURKA. Our previous findings had established that DSF downregulates AURKA expression and alters autophagy-related markers in the context of MASH. However, whether AURKA inhibition is essential for these beneficial effects or merely a correlated event remained unclear. The current data address this critical gap. We demonstrated that pharmacological activation of AURKA, at a non-toxic concentration (60 µM), significantly reversed DSF-mediated reductions in lipid accumulation (Figure 7B,C) and abolished the changes in autophagy-related markers, including TIM23 and TOMM40, alongside re-accumulation of the autophagy substrate P62 (Figure 7D). These results provide the first direct pharmacological evidence that inhibiting AURKA is not only associated with but is also mechanistically necessary for DSF’s ability to alleviate lipotoxicity and alter autophagy-related markers in hepatocytes.

Several limitations of this study should be acknowledged. First, the CDAA diet model, while effectively inducing steatosis, inflammation, and fibrosis, does not fully recapitulate the metabolic milieu of human MASH, particularly obesity and insulin resistance, which may limit the translatability of our findings. Second, we observed a difference in body weight trajectories between groups (Figure 3B), and we cannot exclude the possibility that weight change confounded the protective effects of DSF. Future pair-feeding or metabolic studies are needed to address this. Second, this study used only male mice, a single DSF dose (25 mg/kg), and one time point (4 weeks, *n* = 5 per group). Given sex differences in MASH pathogenesis, the efficacy of DSF in female animals and the dose–response and long-term effects remain to be determined. Third, although we identified AURKA as a key node mediating DSF effects, the precise mechanism—whether DSF directly inhibits AURKA or indirectly modulates its expression—remains unclear. Further binding assays and pathway studies are needed. Fourth, while our results suggest a correlation between DSF-induced changes in autophagy-related markers and MASH improvement, the causal role of autophagy requires validation. Whether changes in autophagy-related markers are necessary for DSF efficacy or merely a bystander effect should be addressed using hepatocyte-specific autophagy gene knockout models. Finally, although our experiments using an AURKA activator confirm that disulfiram’s hepatoprotective effect critically depends on AURKA inhibition, the precise molecular interaction—whether disulfiram directly binds AURKA or acts through upstream regulators—remains to be fully elucidated and warrants further investigation. Despite these limitations, our findings provide a strong proof-of-concept for DSF as a candidate for MASH intervention, and future studies with improved designs are warranted.

## 5. Conclusions

**Figure 8:**
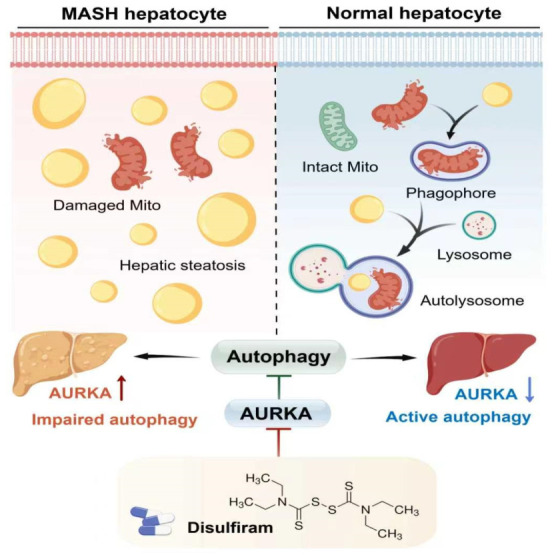
Mechanism diagram: DSF alleviates MASH by specifically inhibiting AURKA in MASH hepatocytes. Elevated AURKA levels are associated with reduced autophagy-related markers, including mitophagy-related markers, leading to damaged mitochondria, hepatic steatosis, and lipotoxicity. By inhibiting AURKA, DSF is associated with changes in autophagy-related markers, enhances the clearance of lipid droplets and damaged mitochondria, and reduces lipotoxicity.

In conclusion, this study demonstrates that DSF alleviates CDAA diet-induced MASH-associated liver injury and lipotoxicity through mechanisms involving AURKA downregulation and changes in autophagy-related markers (Figure 8). More importantly, we identify the AURKA–autophagy axis as a novel intervention node in metabolic disease, enriching the non-canonical functions of AURKA and providing a conceptual framework for drug repurposing in complex metabolic disorders.

## Figures and Tables

**Table 1:** The primer sequence of real-time PCR.

Gene	Gene Accession Number	Forward (5′-3′)	Reverse (5′-3′)
SREBP1c	NM_011480.4	5′-GGTACCTGCGGGACAGCTTA-3′	5′-CCGTGAGCTACCTGGACTGAA-3′
ACCα	NM_133360.3	5′-CAAGCCCGTGAGAACACAG-3′	5′-GAGTGTGTTTGACCAGGAACAATGA-3′
FASN	NM_007988.3	5′-GGAGGTGGTGATAGCCGGTAT-3′	5′-TGGGTAATCCATAGAGCCCAG-3′
CPT-1A	NM_013495.2	5′-GATCAATCGGACCCTAGACAC-3	5′-AGAGCAGCACCTTCAGCGAGTA-3
LOX	NM_010728.3	5′-TCTTCTGCTGCGTGACAACC-3′	5′-GAGAAACCAGCTTGGAACCAG-3′
TGF-β	NM_011577.2	5′-CTCCCGTGGCTTCTAGTGC-3′	5′-GCCTTAGTTTGGACAGGATCTG-3′
CCL2	NM_011333.3	5′-AGCAGCAGGTGTCCCAAAGA-3′	5′-GTGCTGAAGACCTTAGGGCAGA-3′
TNF-α	NM_013693.3	5′-CAGGCGGTGCCTATGTCTC-3′	5′-CGATCACCCCGAAGTTCAGTAG-3′
CXCL2	NM_009140.2	5′-GCCAAGGGTTGACTTCAAGAACA-3′	5′-AGGCTCCTCCTTTCCAGGTCA-3′
AURKA	NM_011497.4	5′-AGACAAAGCAAGTTCATCCTGG-3′	5′-TGTTCCAAGGGGCGCATATTC-3′
APOB	NM_009693.4	5′-AAGCACCTCCGAAAGTACGTG-3′	5′-CGGGAGCGACACCATTTACAA-3′
GAPDH	NM_008084.3	5′-AGGTCGGTGTGAACGGATTTG-3′	5′-TGTAGACCATGTAGTTGAGGTCA-3′

SREBP1c, sterol regulatory element binding transcription factor 1; ACCα, acetyl-CoA carboxylase alpha; FASN, fatty acid synthase; CPT-1A, carnitine palmitoyltransferase 1A; LOX, lysyl oxidase; TGF-β, transforming growth factor beta; TNF-α, tumor necrosis factor-α; CXCL2, C-X-C motif chemokine ligand 2; AURKA, aurora kinase A; APOB, apolipoprotein B; GAPDH, glyceraldehyde-3-phosphate dehydrogenase.

## Data Availability

All data are included in this manuscript.

## Supplementary Materials

The following supporting information can be downloaded at: https://www.mdpi.com/article/10.3390/antiox15070867/s1. Figure S1. (A) Representative H&E staining of liver tissue sections from ND mice treated with Vehicle or DSF. (B) Serum ALT levels from each group. (C) Serum AST levels from each group. Data are presented as mean ± SD. ns (not significant) vs. indicated groups.; Figure S2. Relative mRNA expression of AURKA in liver tissue across ND, CDAA, and CDAA+DSF groups. Data are presented as mean ± SD. *** *p* < 0.001 vs. indicated groups.
