# Supplementary material for: Disulfiram Alleviates Metabolic Dysfunction-Associated Steatohepatitis in Mice via Inhibiting Aurora Kinase A and Restoring Autophagy
Source: Antioxidants (Basel). 2026 Jul 11;15(7):867. doi: 10.3390/antiox15070867 (PMC13405444; doi:10.3390/antiox15070867)

Supplementary Figure S1

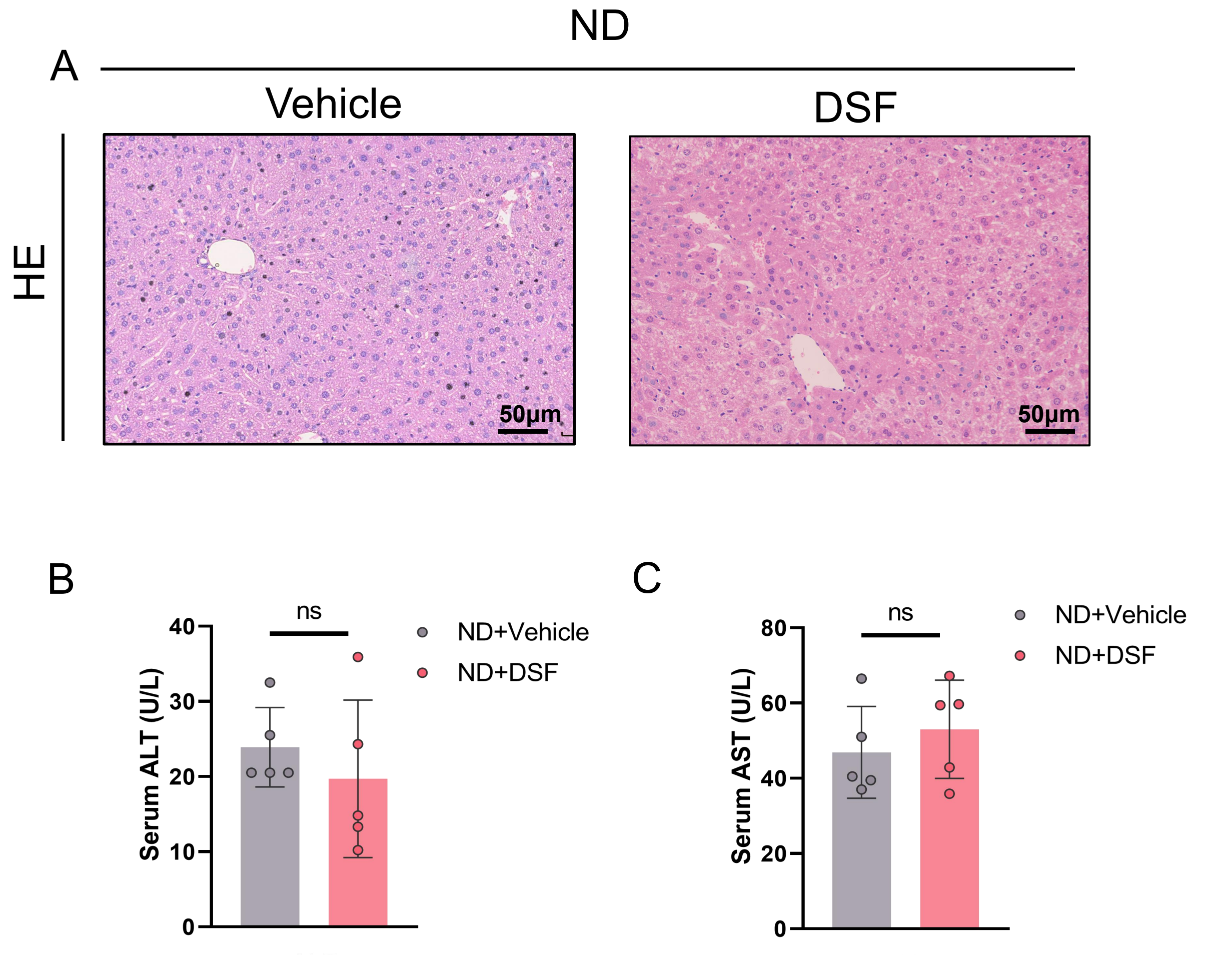

Figure legend: (A) Representative H&E staining of liver tissue sections from ND mice treated with Vehicle or DSF. (B) Serum ALT levels from each group. (C) Serum AST levels from each group. Data are presented as mean ± SD. ns (not significant) vs. indicated groups.

Supplementary Figure S2

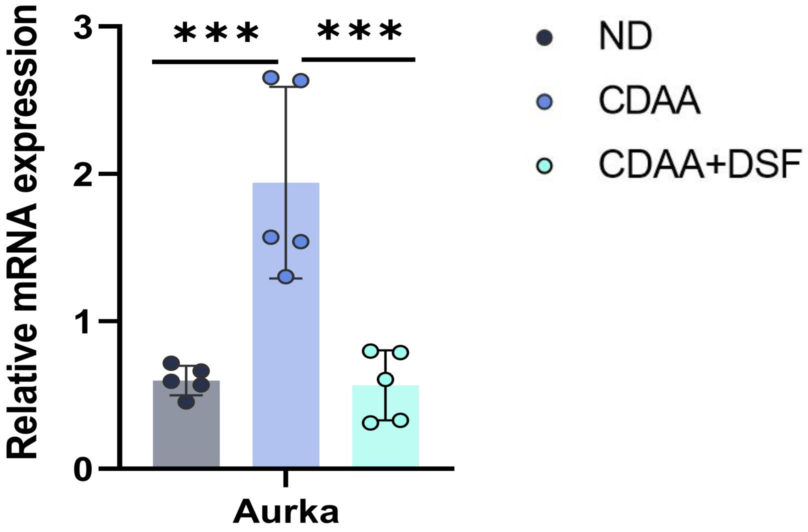

Figure legend: Relative mRNA expression of Aurka in liver tissue across ND, CDAA, and CDAA+DSF groups.Data are presented as mean ± SD. ***$p < 0.001$ vs. indicated groups.

tissue AurKa **Fig 6G**

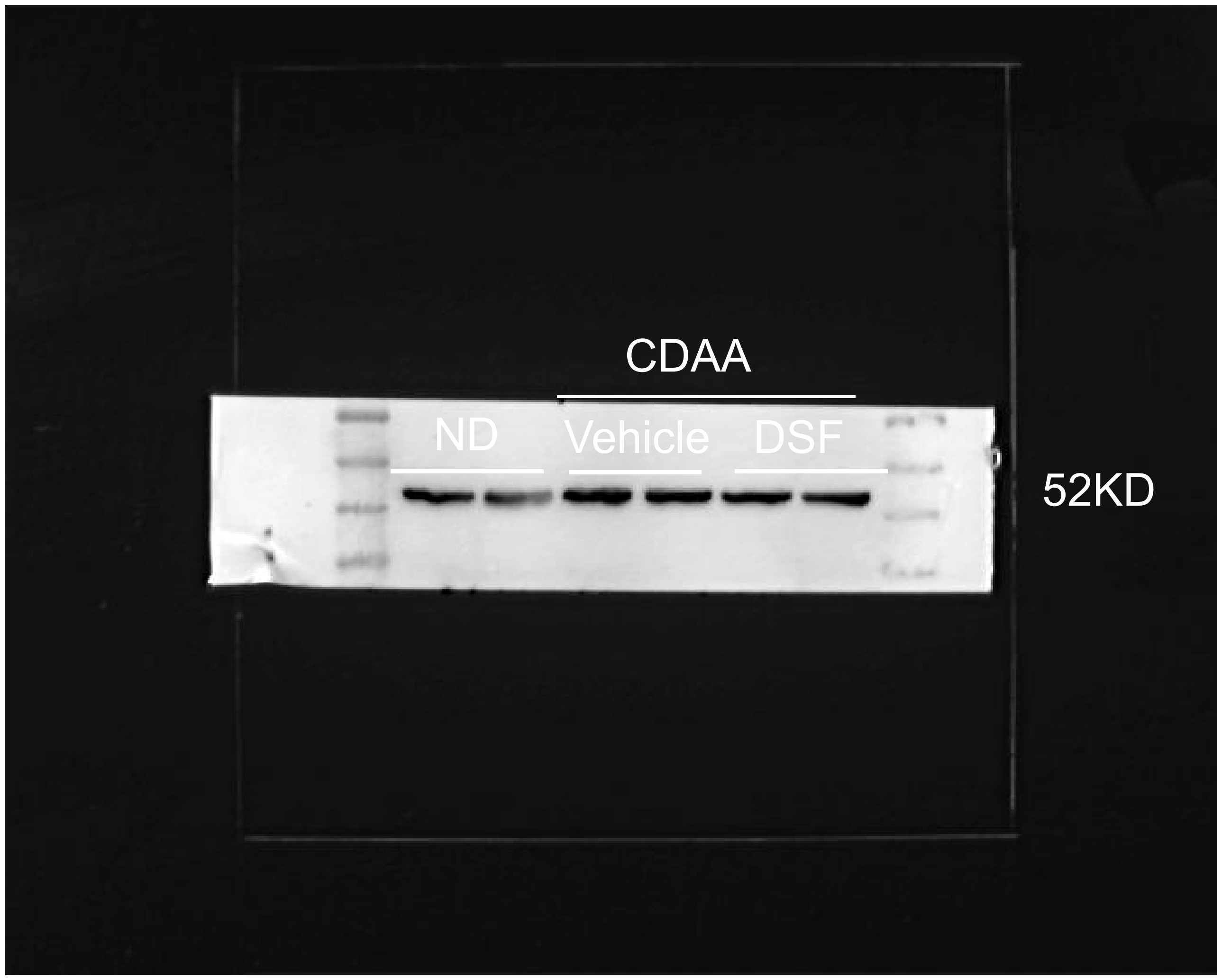

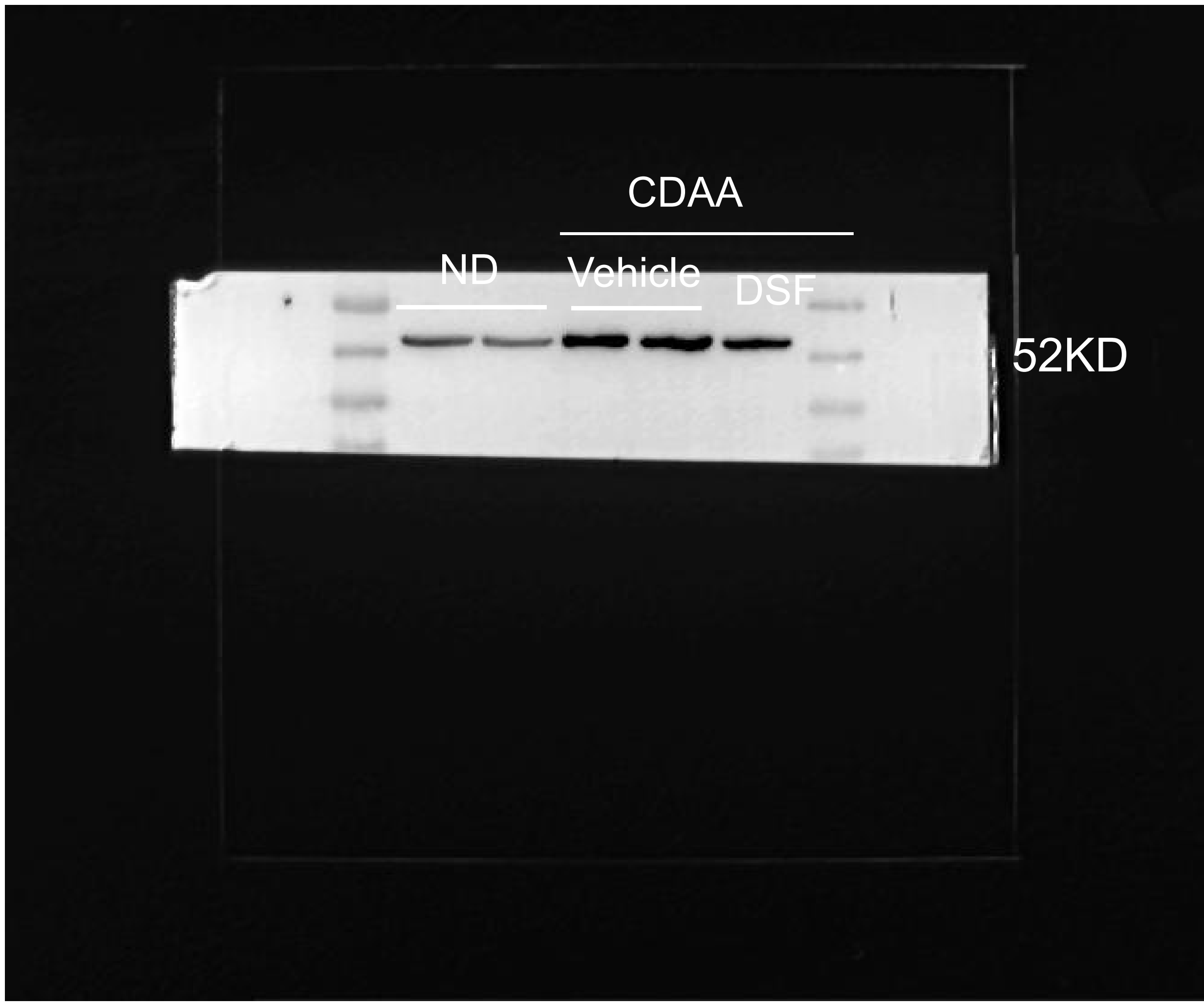

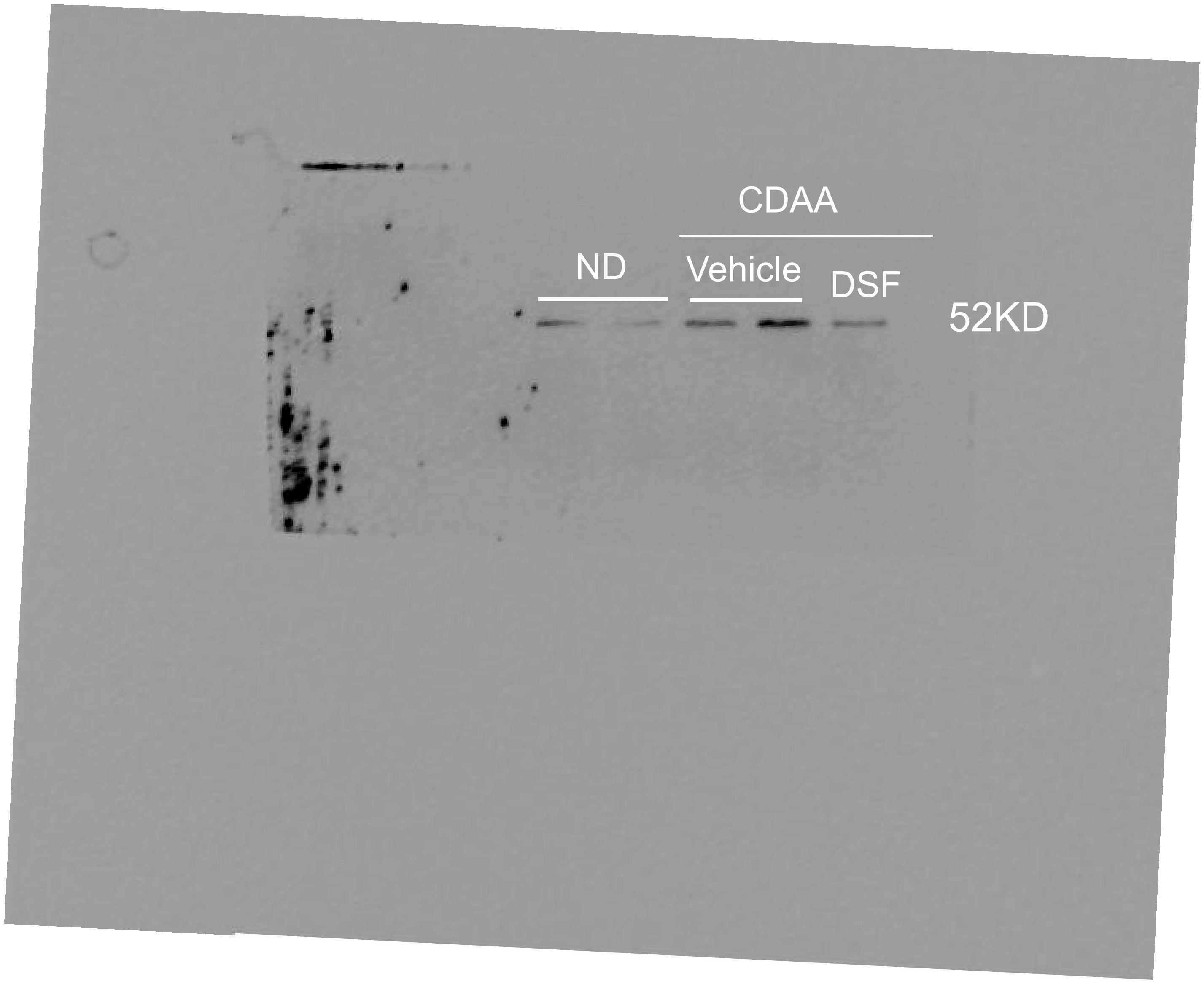

tissue PARK2 Fig 6G

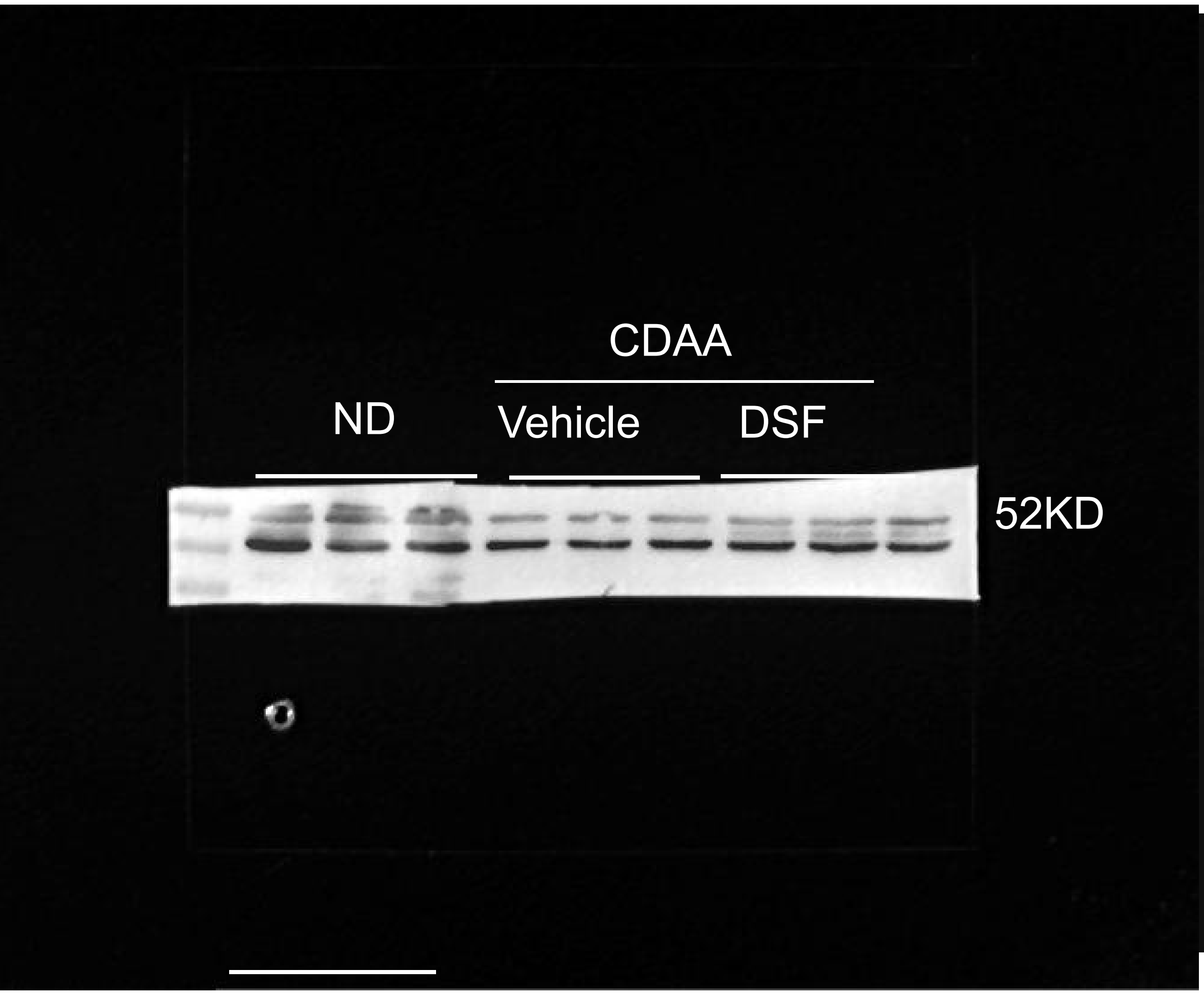

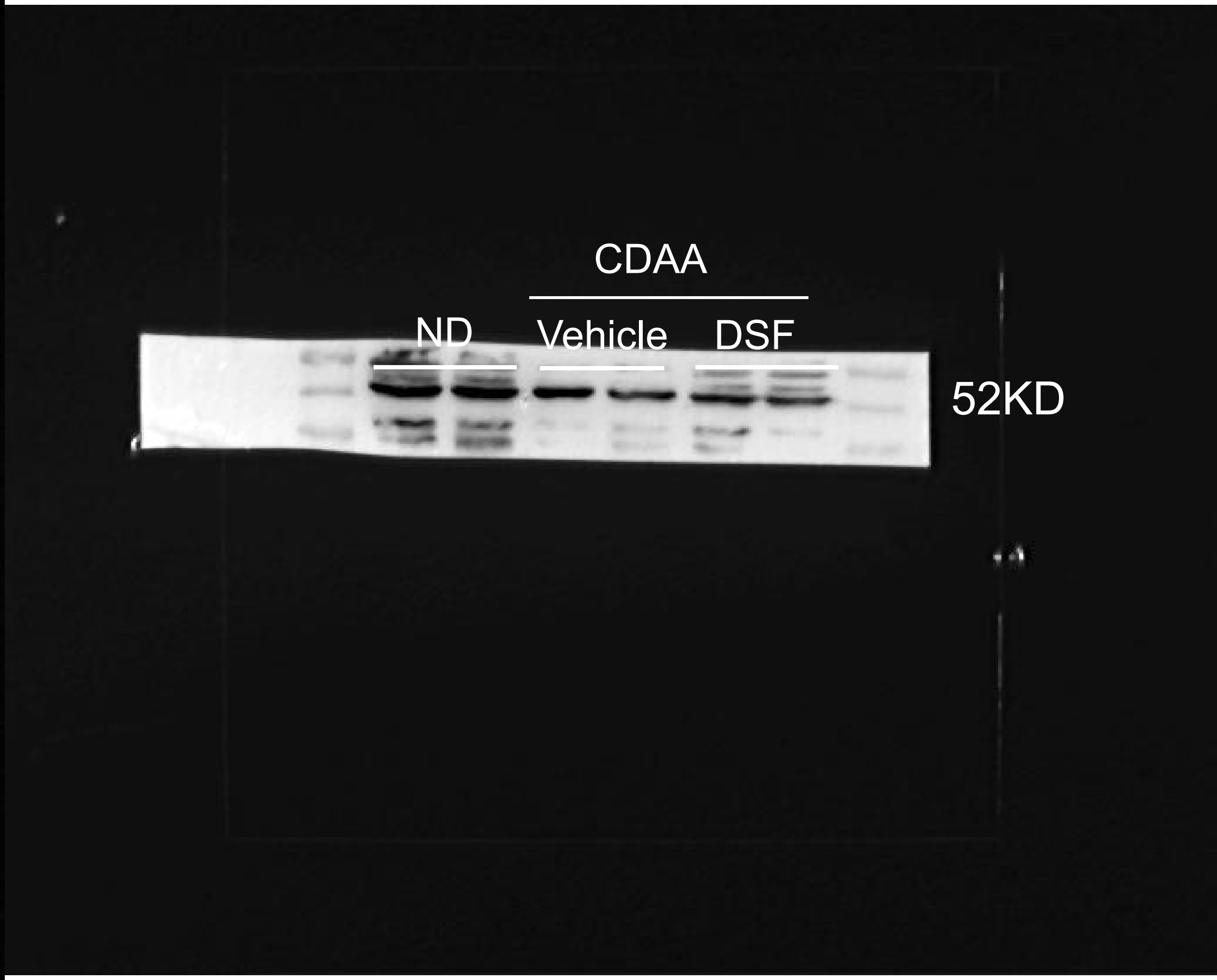

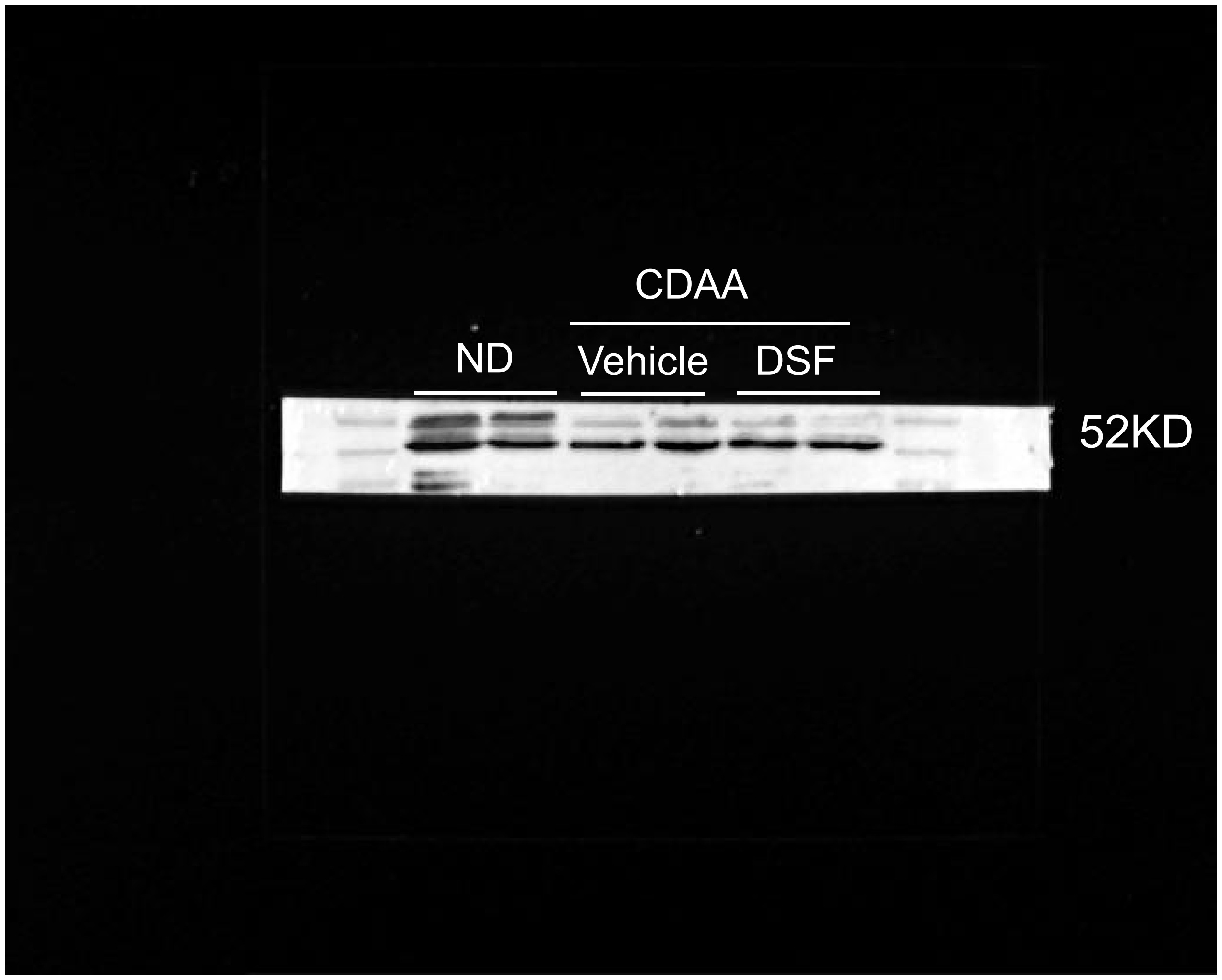

tissue Tim23 Fig 6G

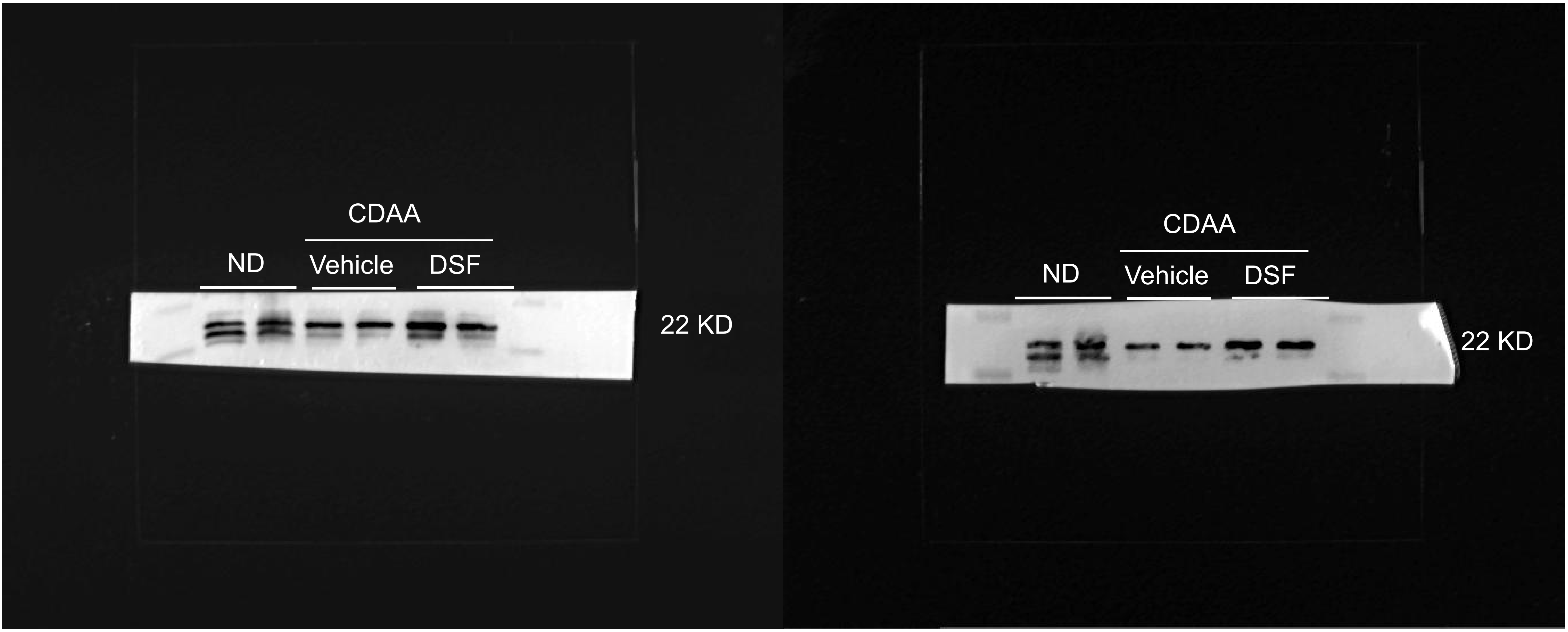

tissue TOMM40 Fig 6G

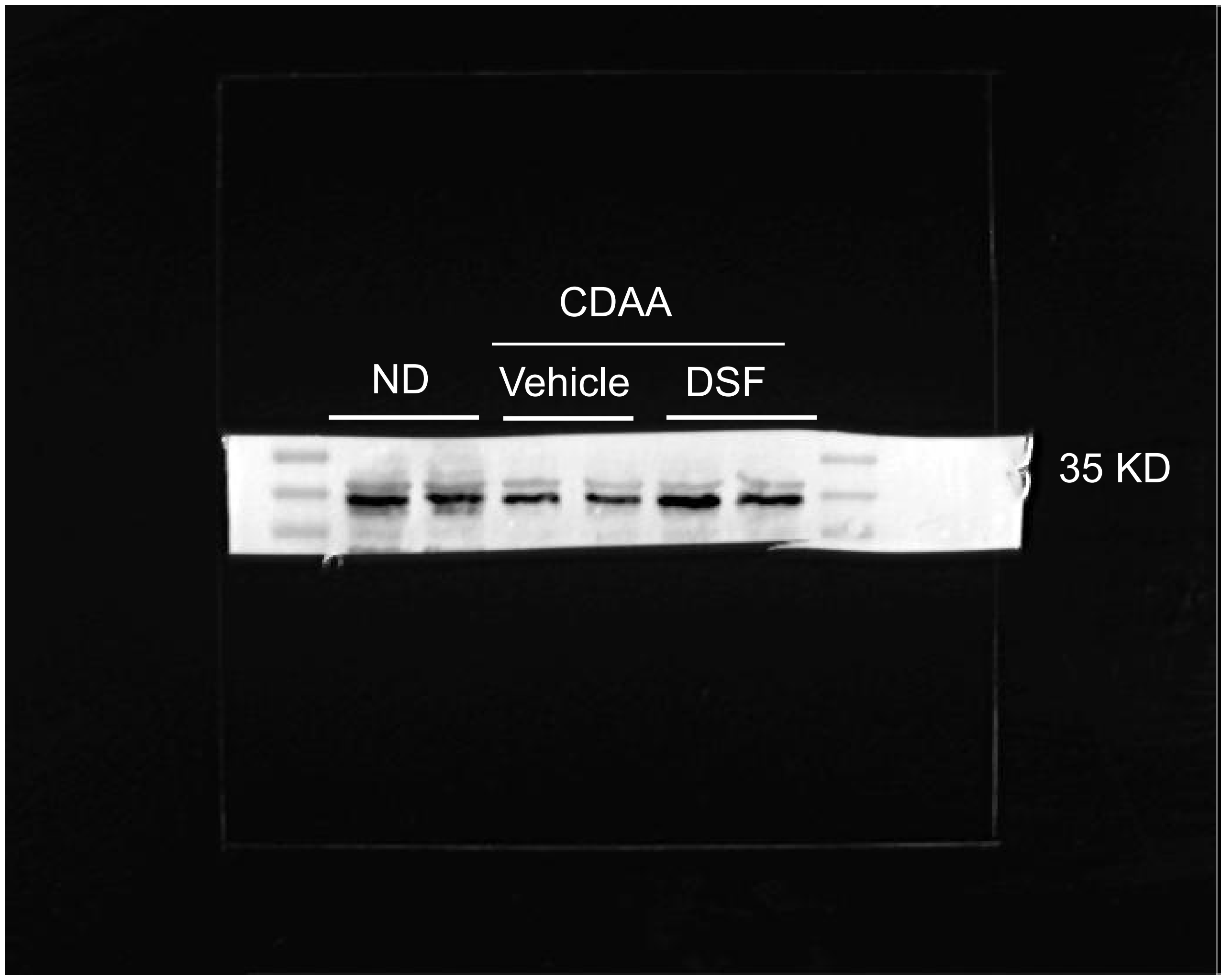

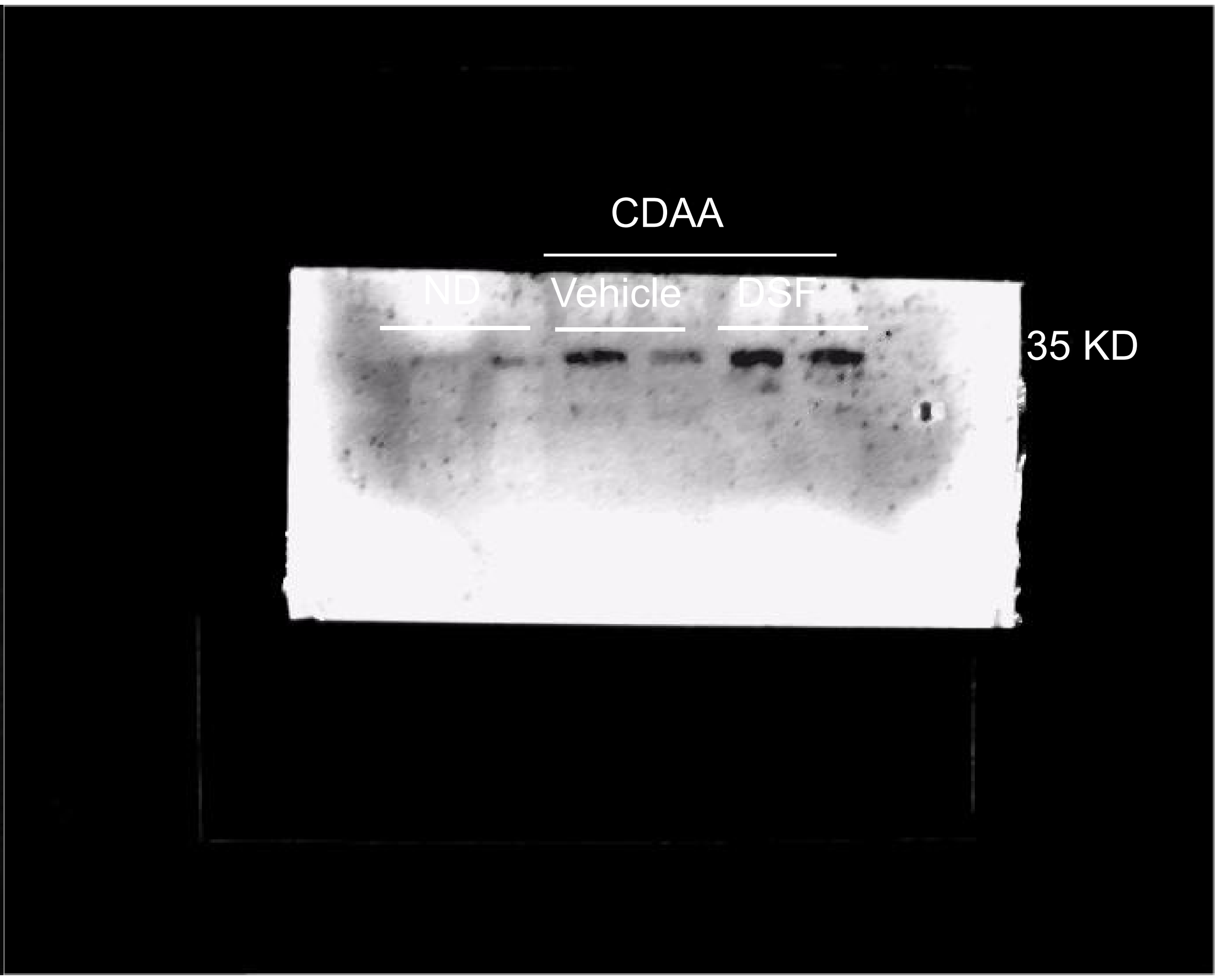

tissue LC3B Fig 6G

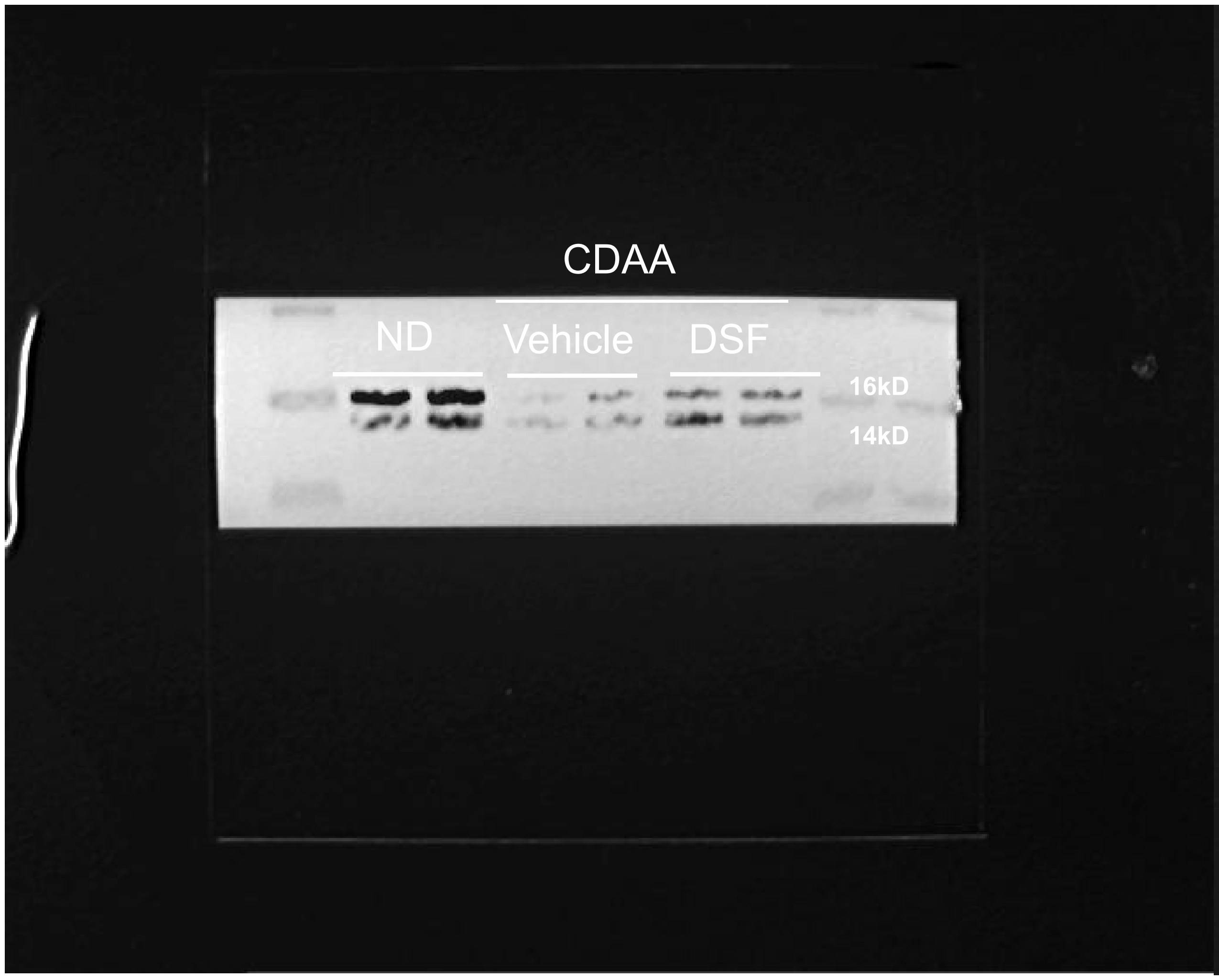

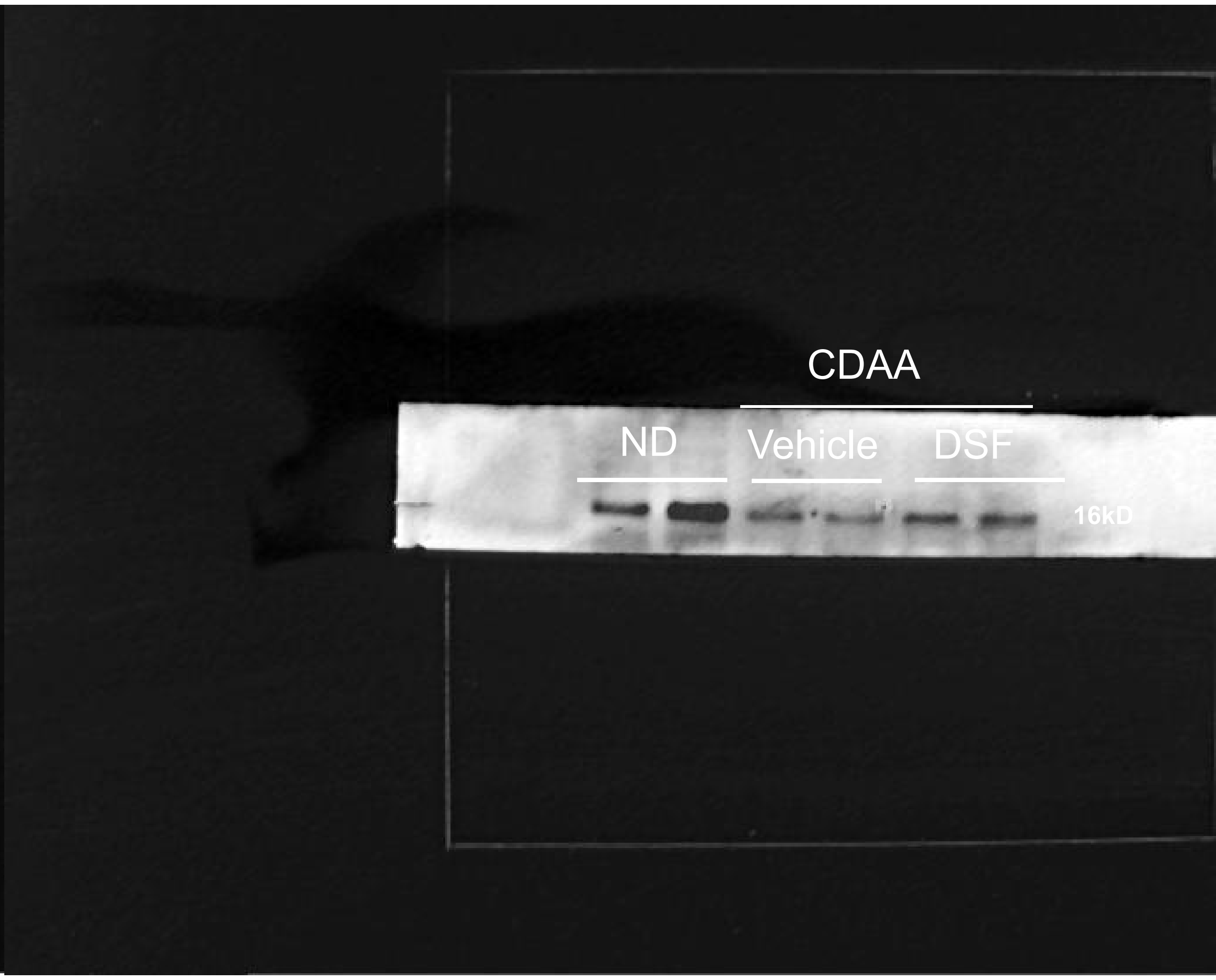

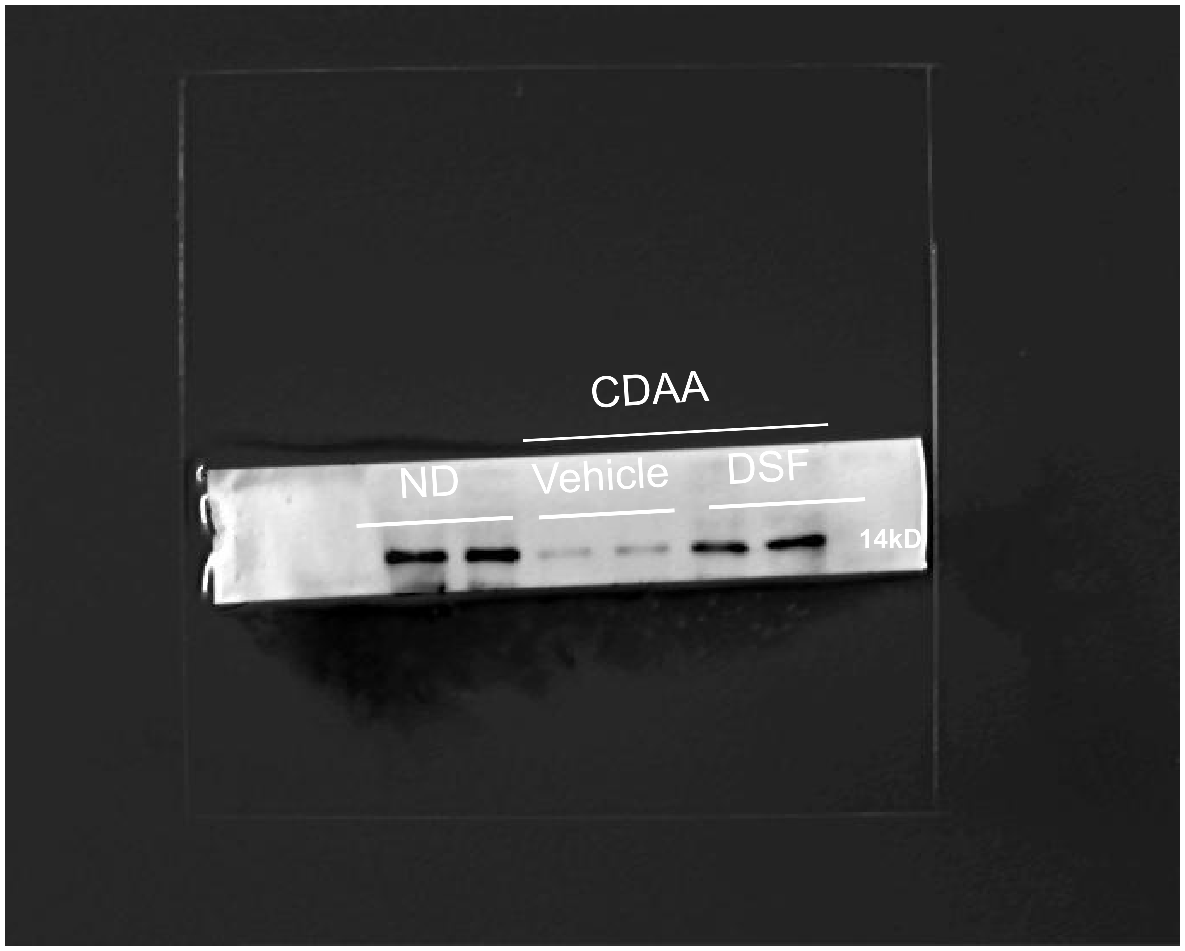

tissue β-actin **Fig 6G**

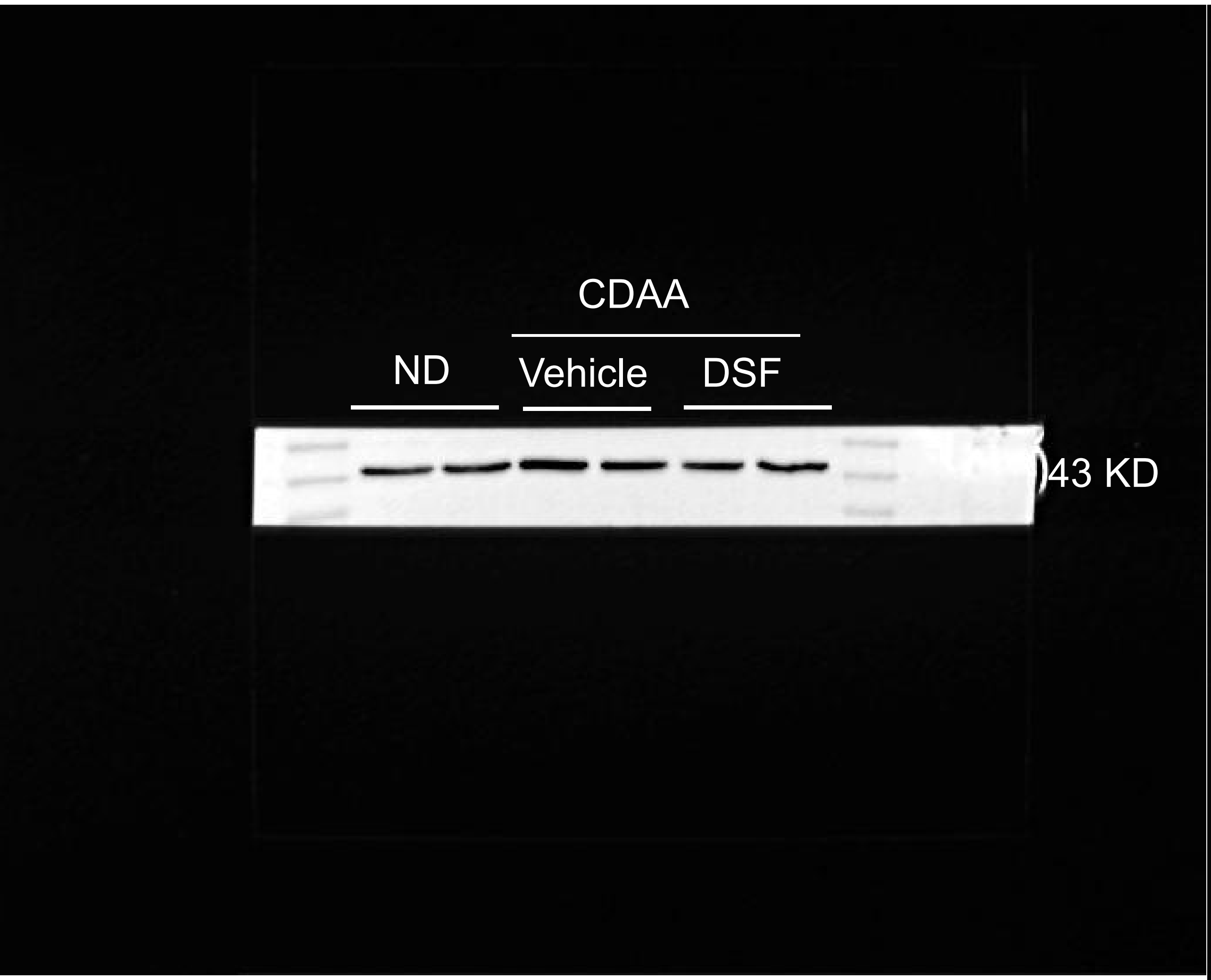

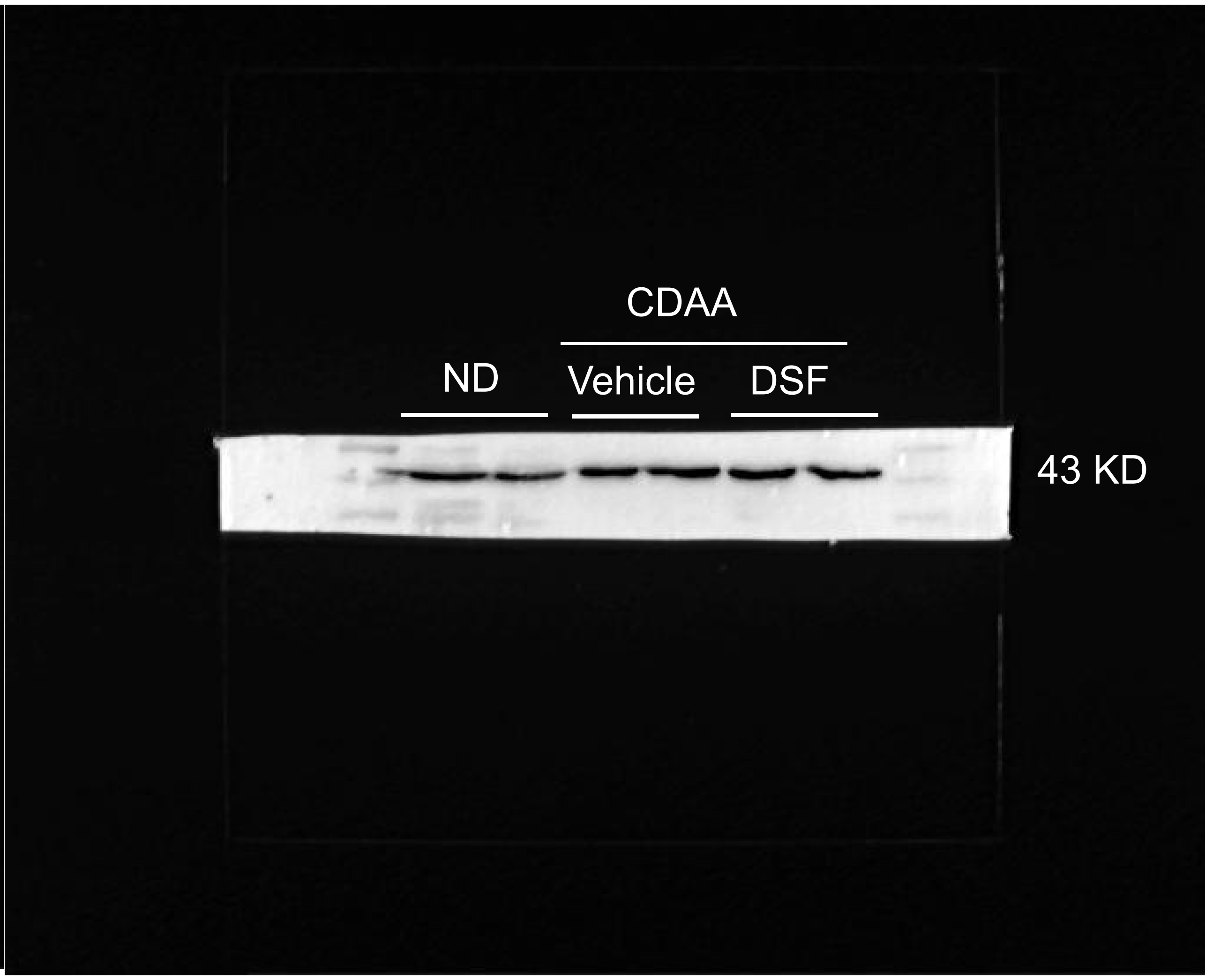

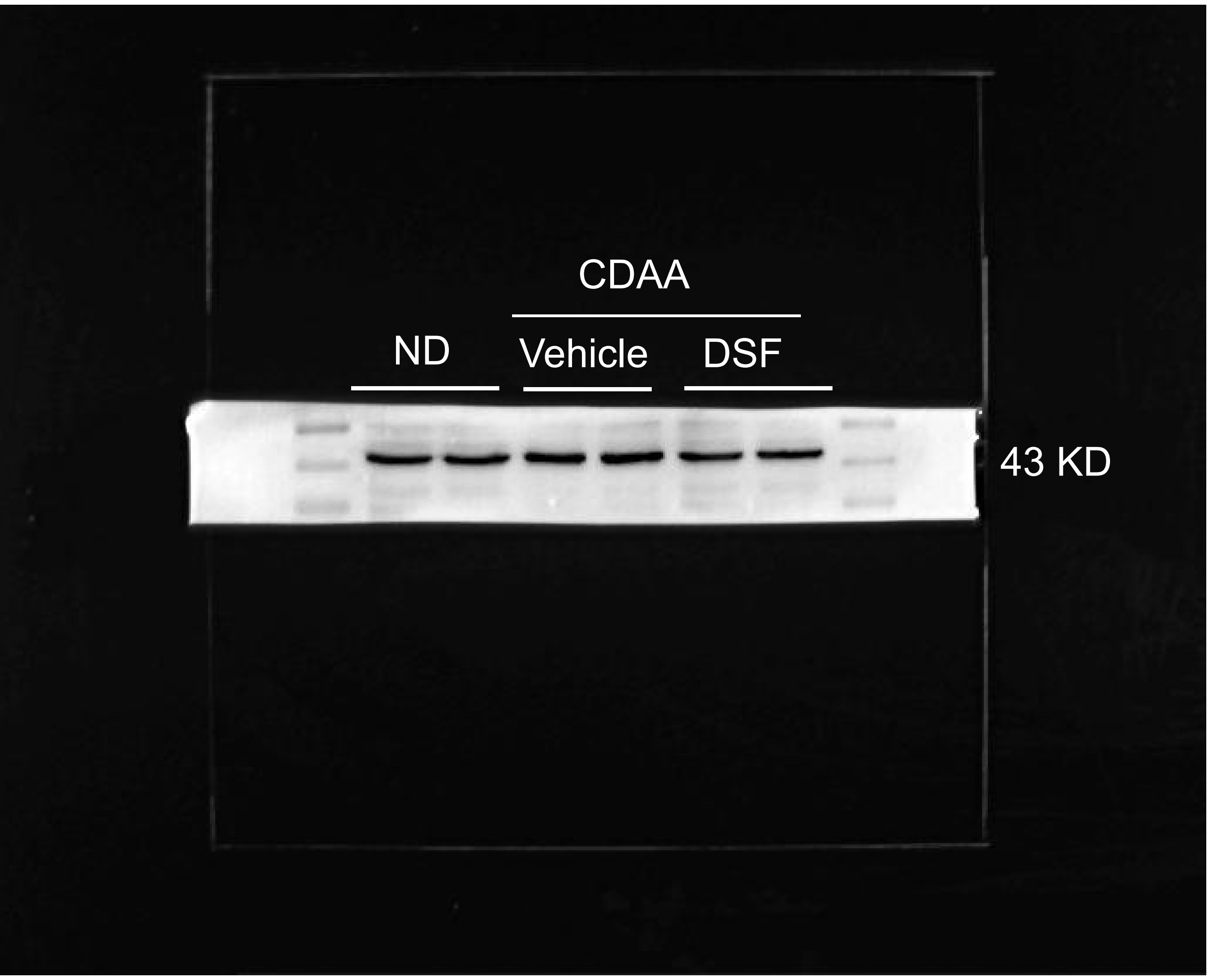

tissue P62   Fig 6G

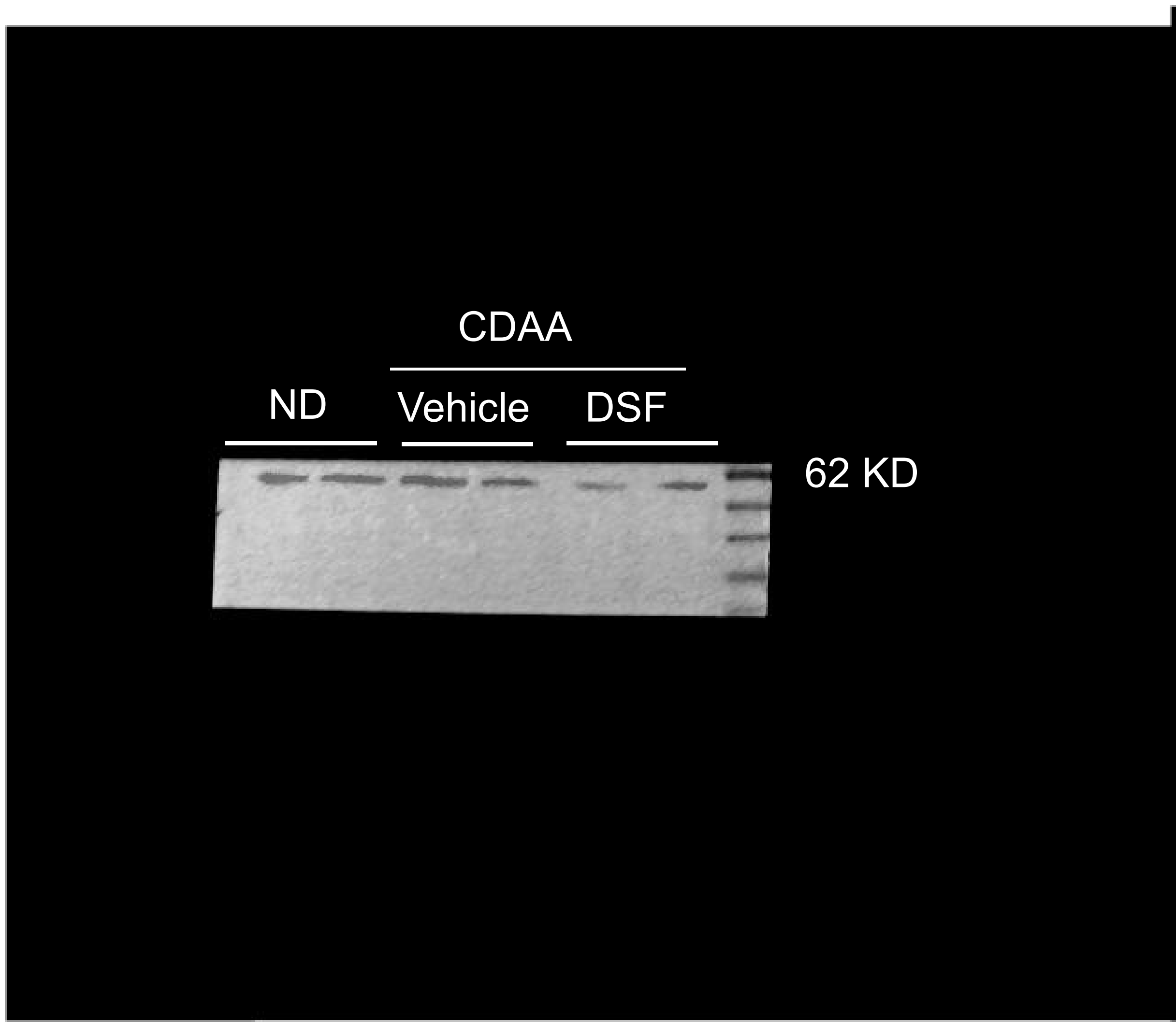

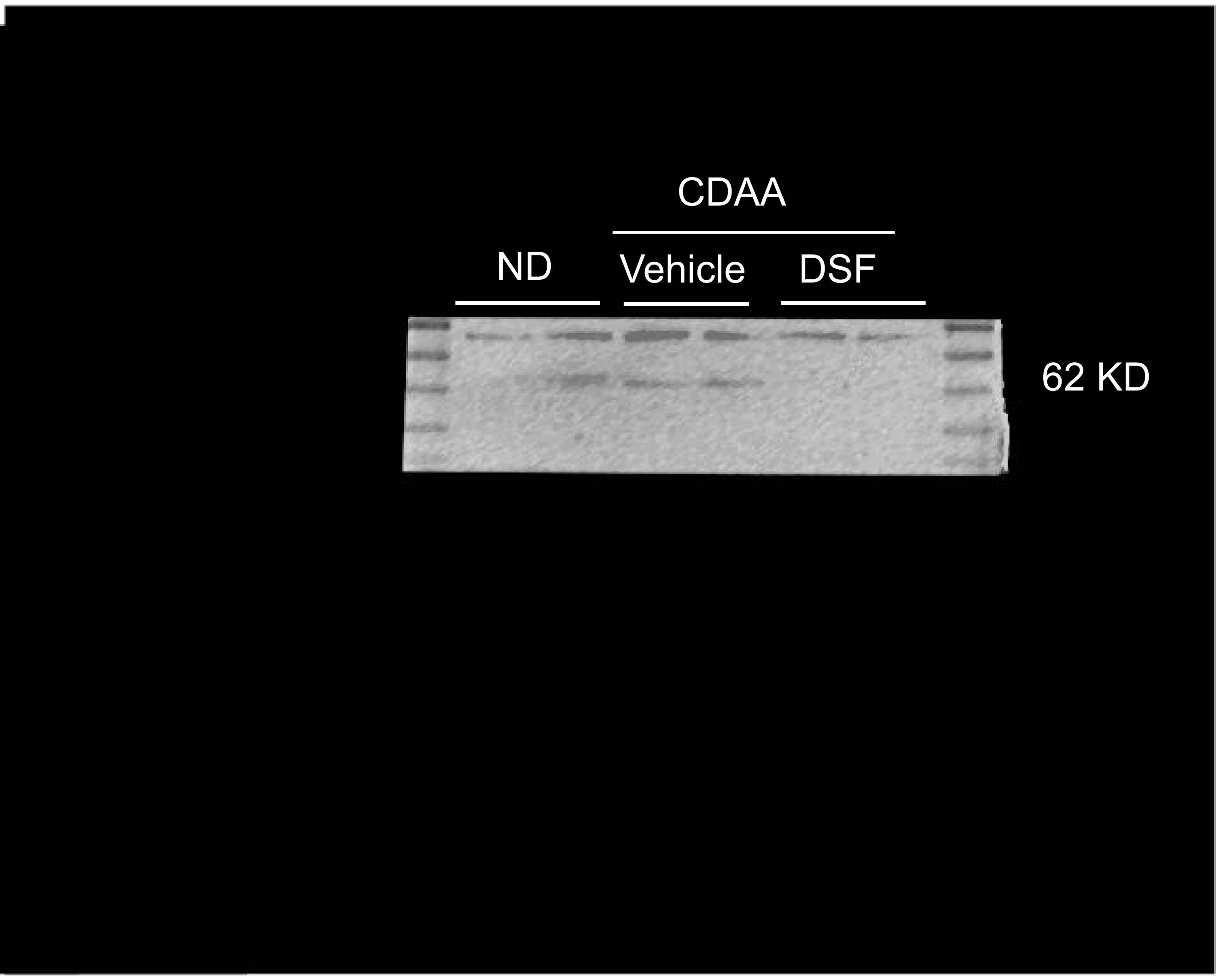

cell AurKa Fig 6I

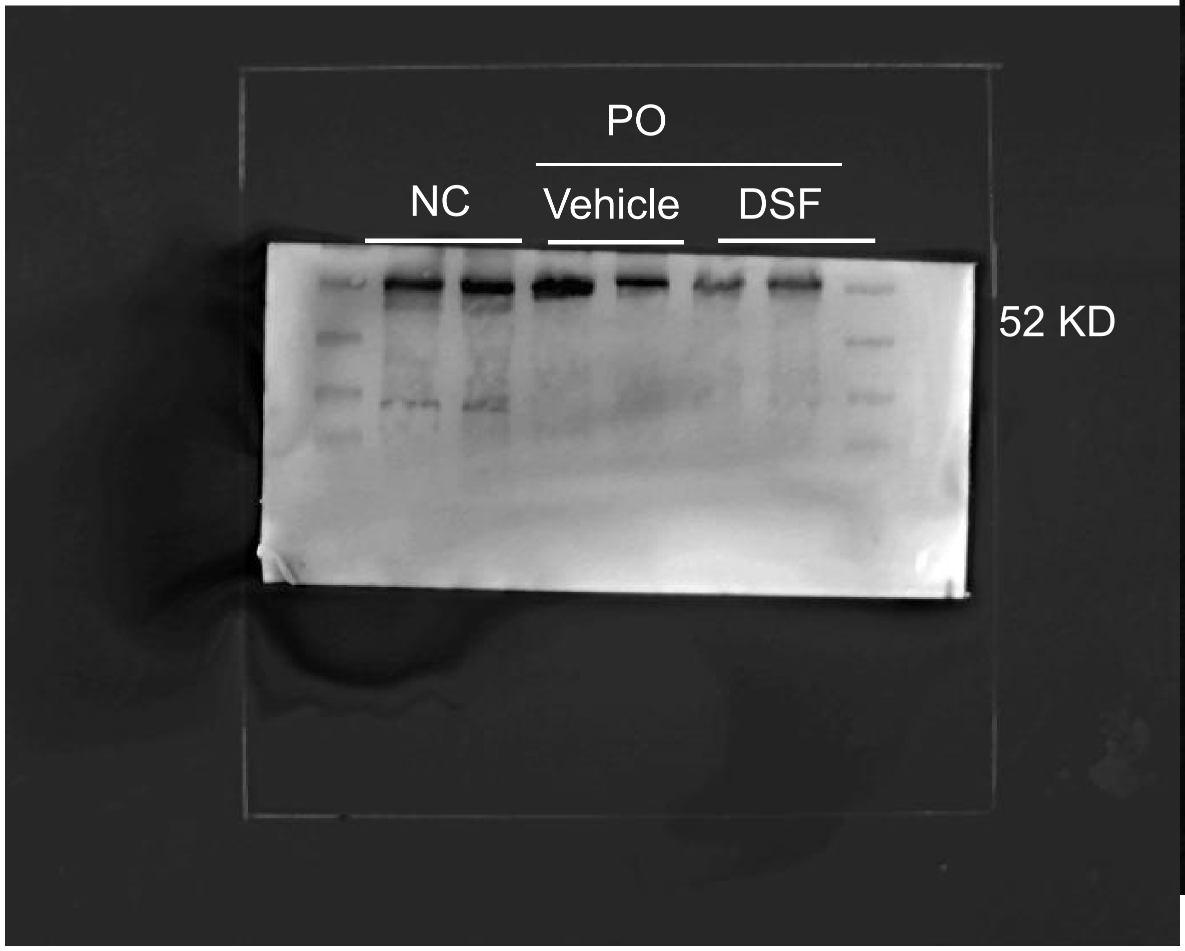

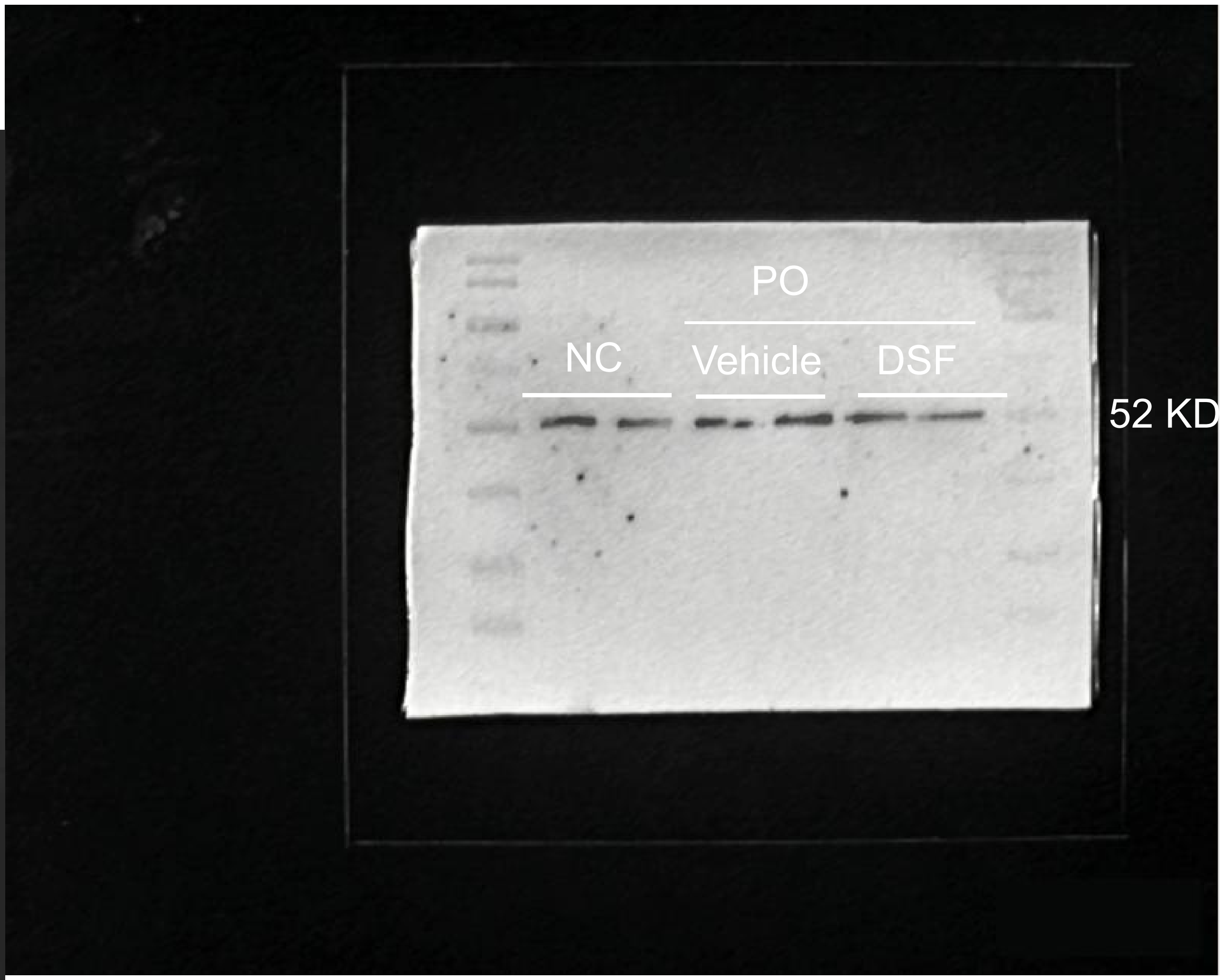

cell β-actin **Fig 6l**

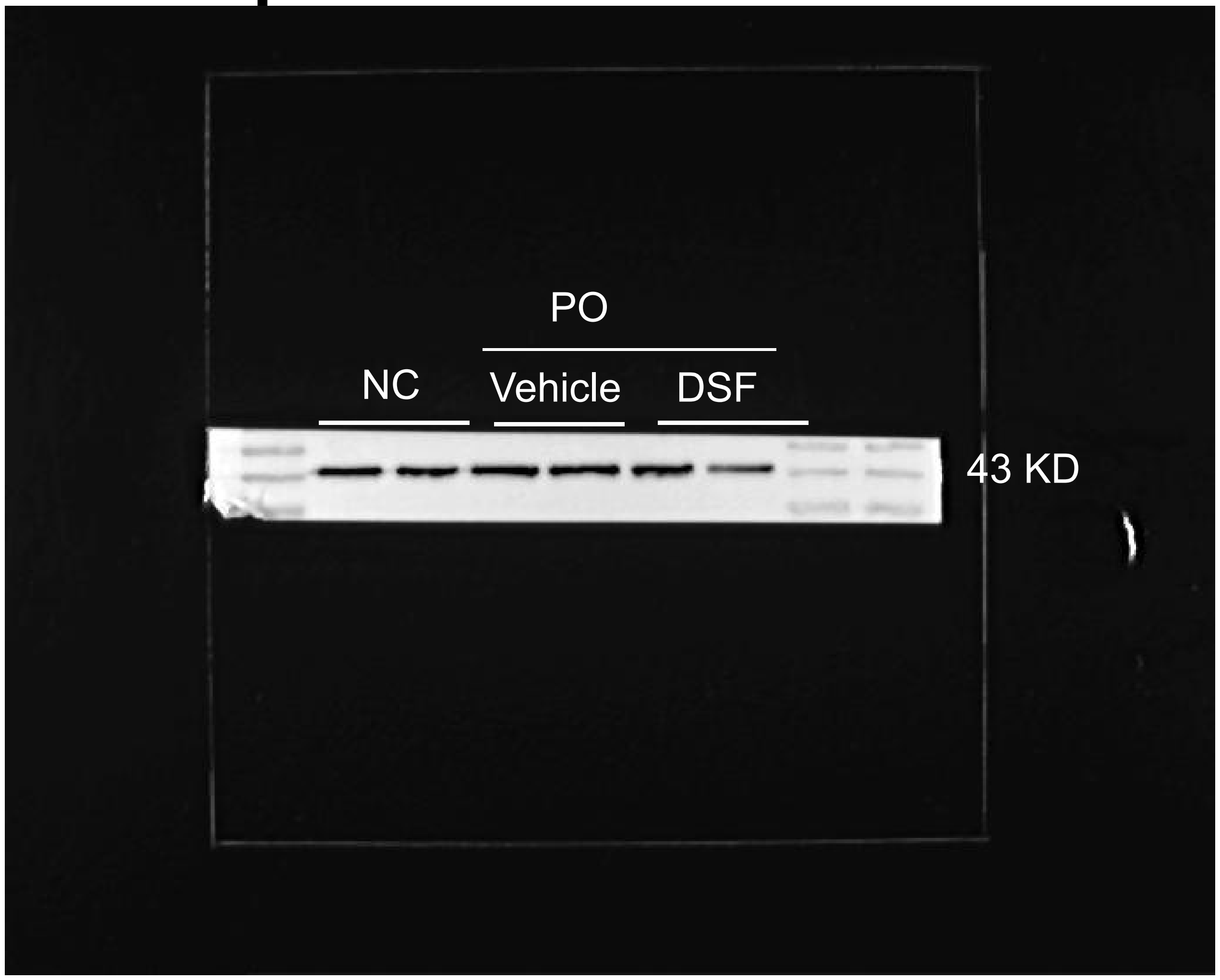

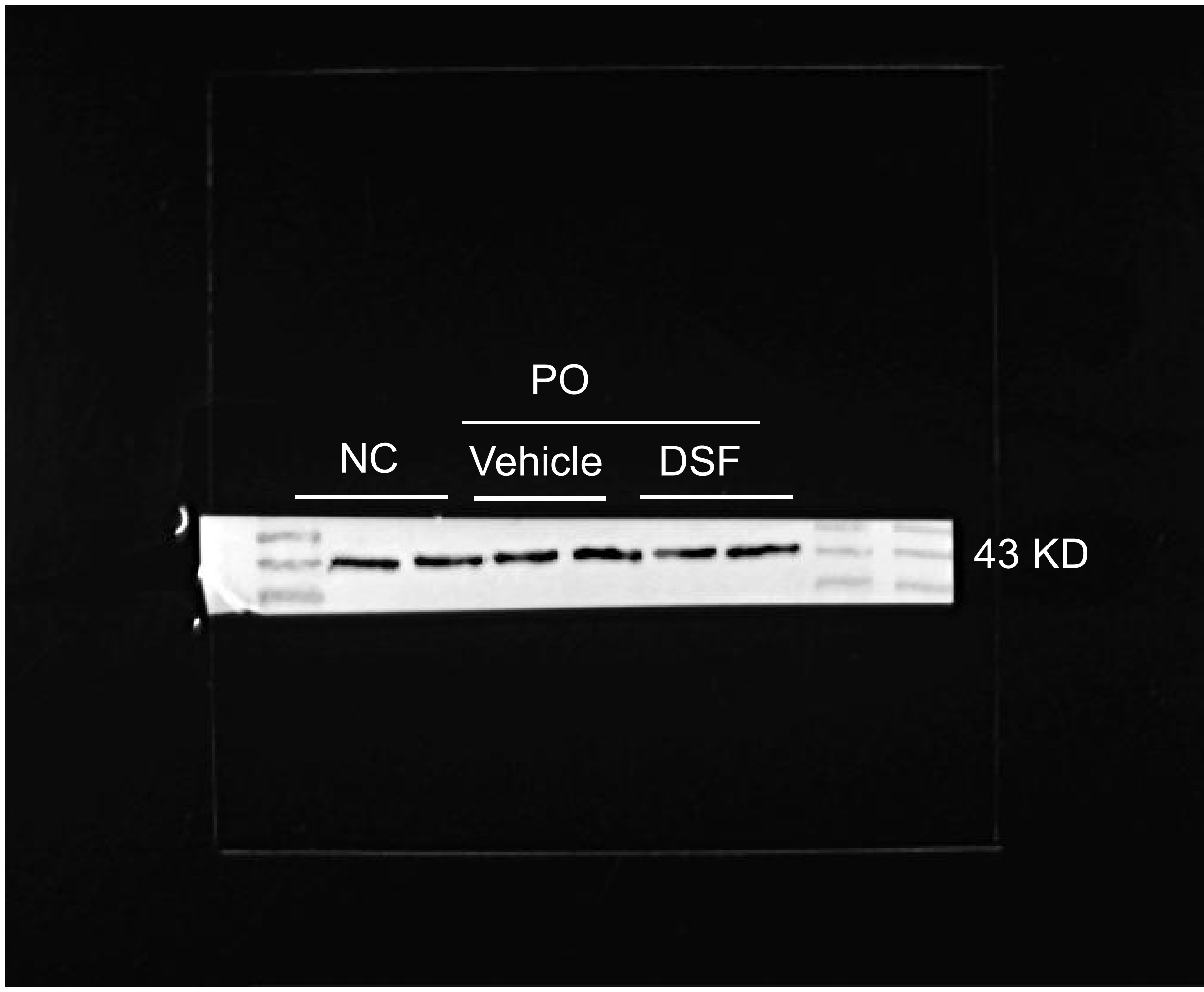

cell Aurka Fig 7D

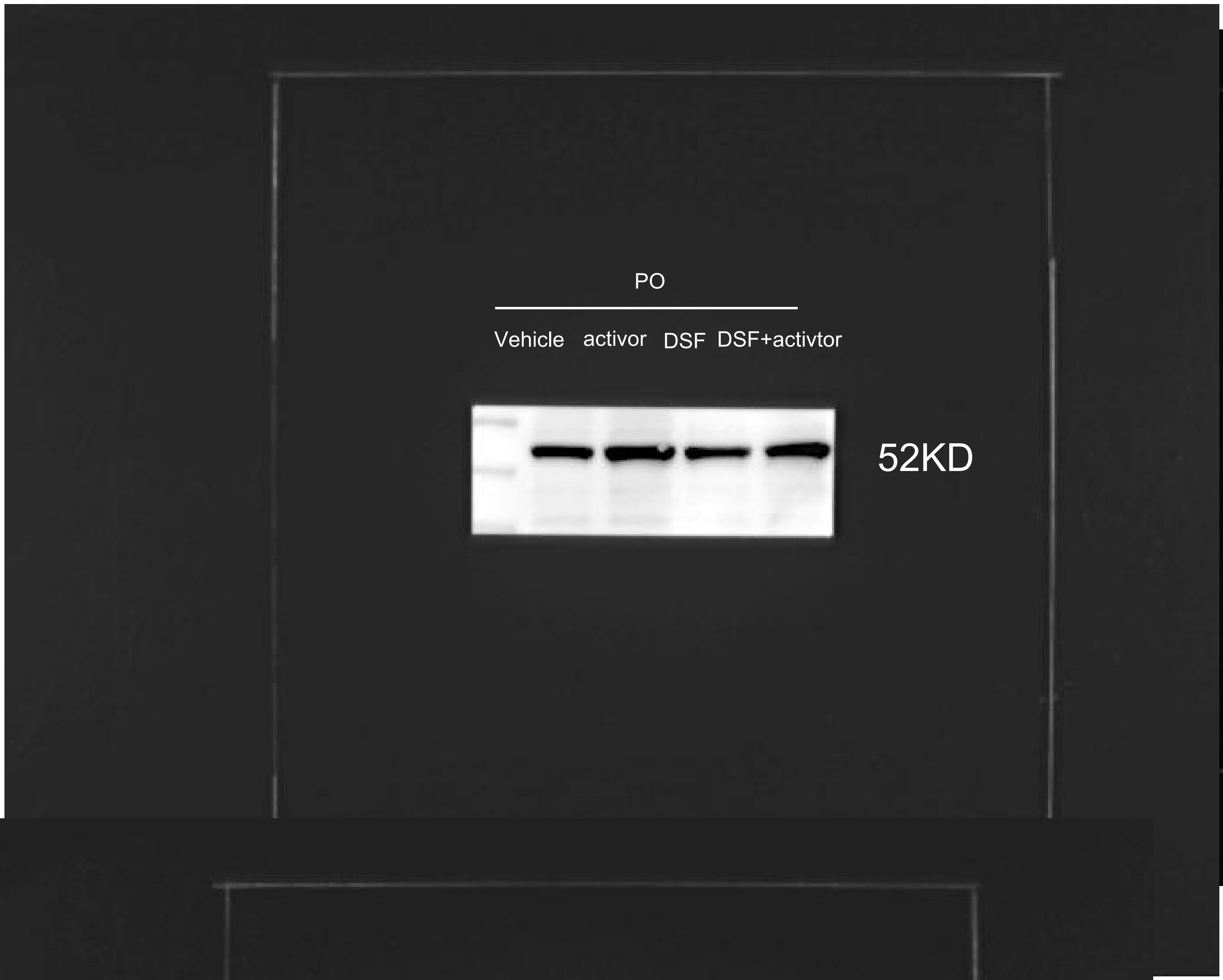

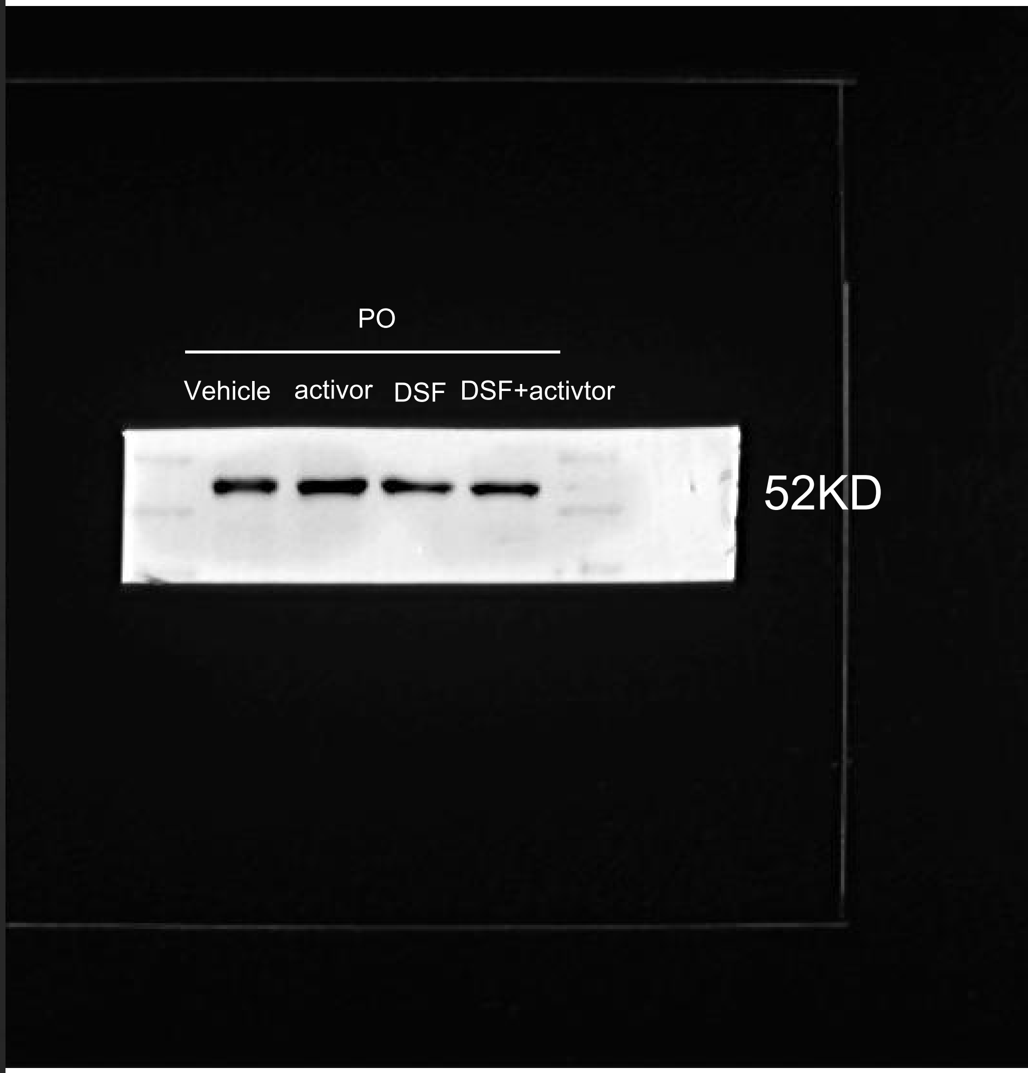

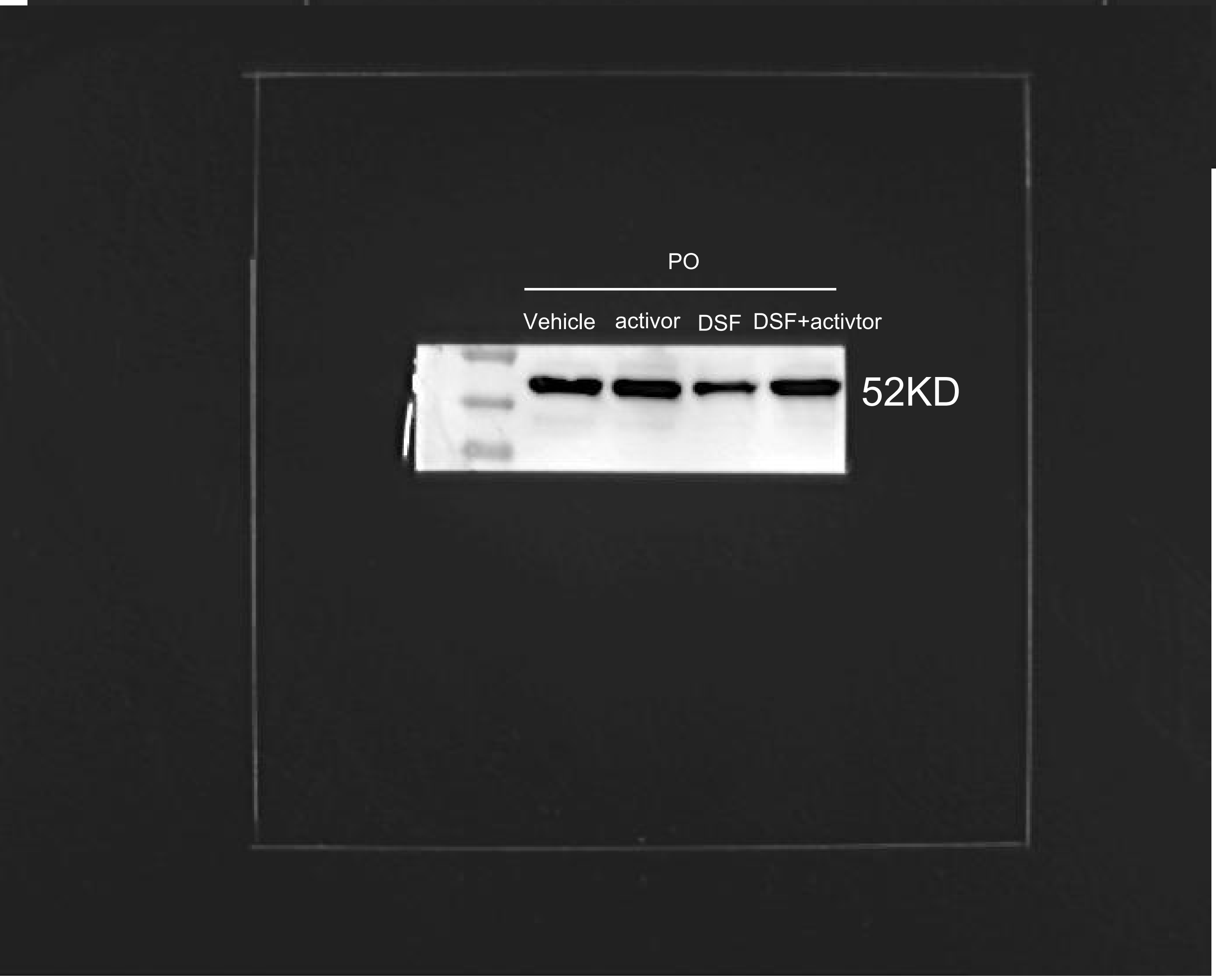

cell tim23 Fig 7D

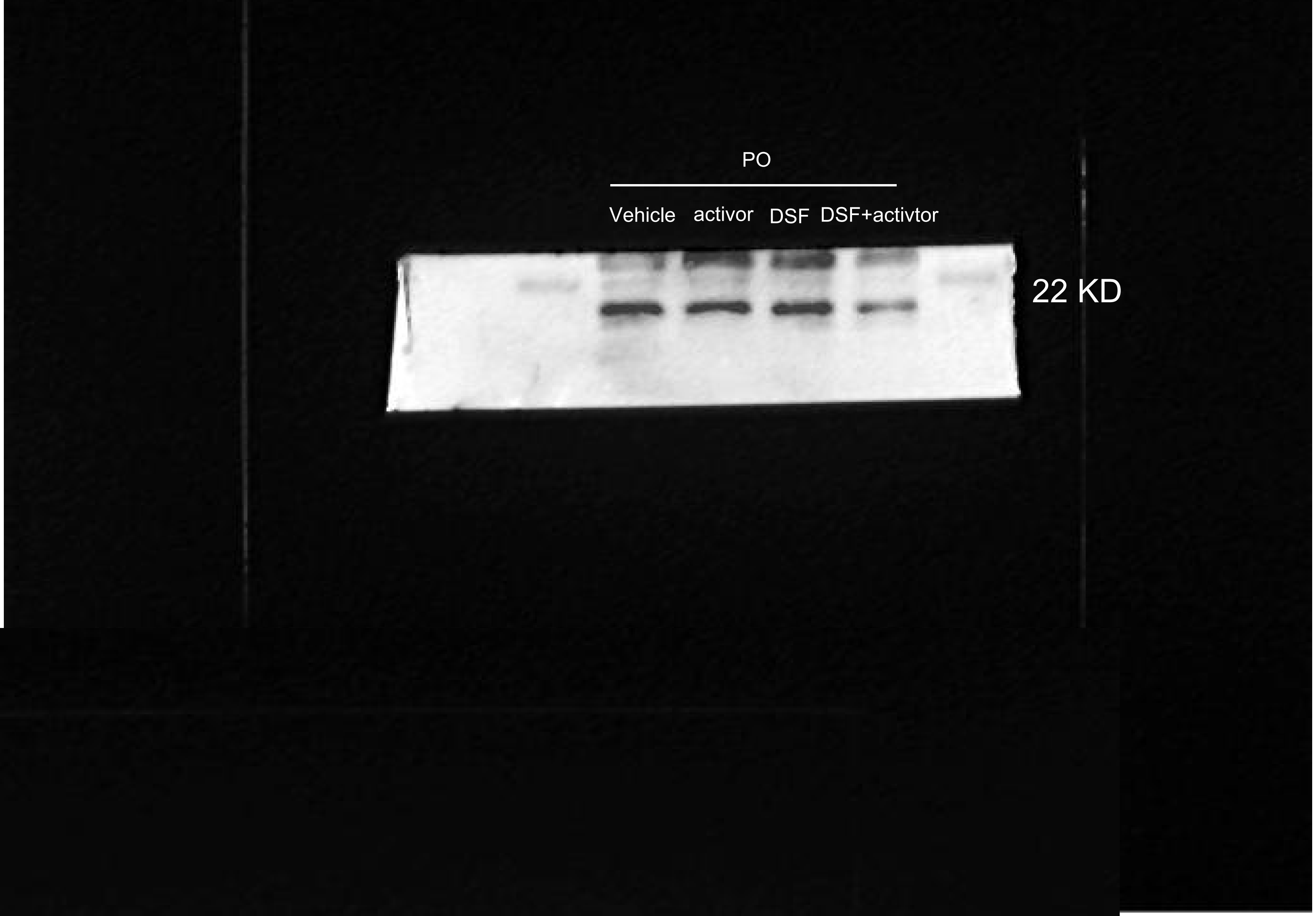

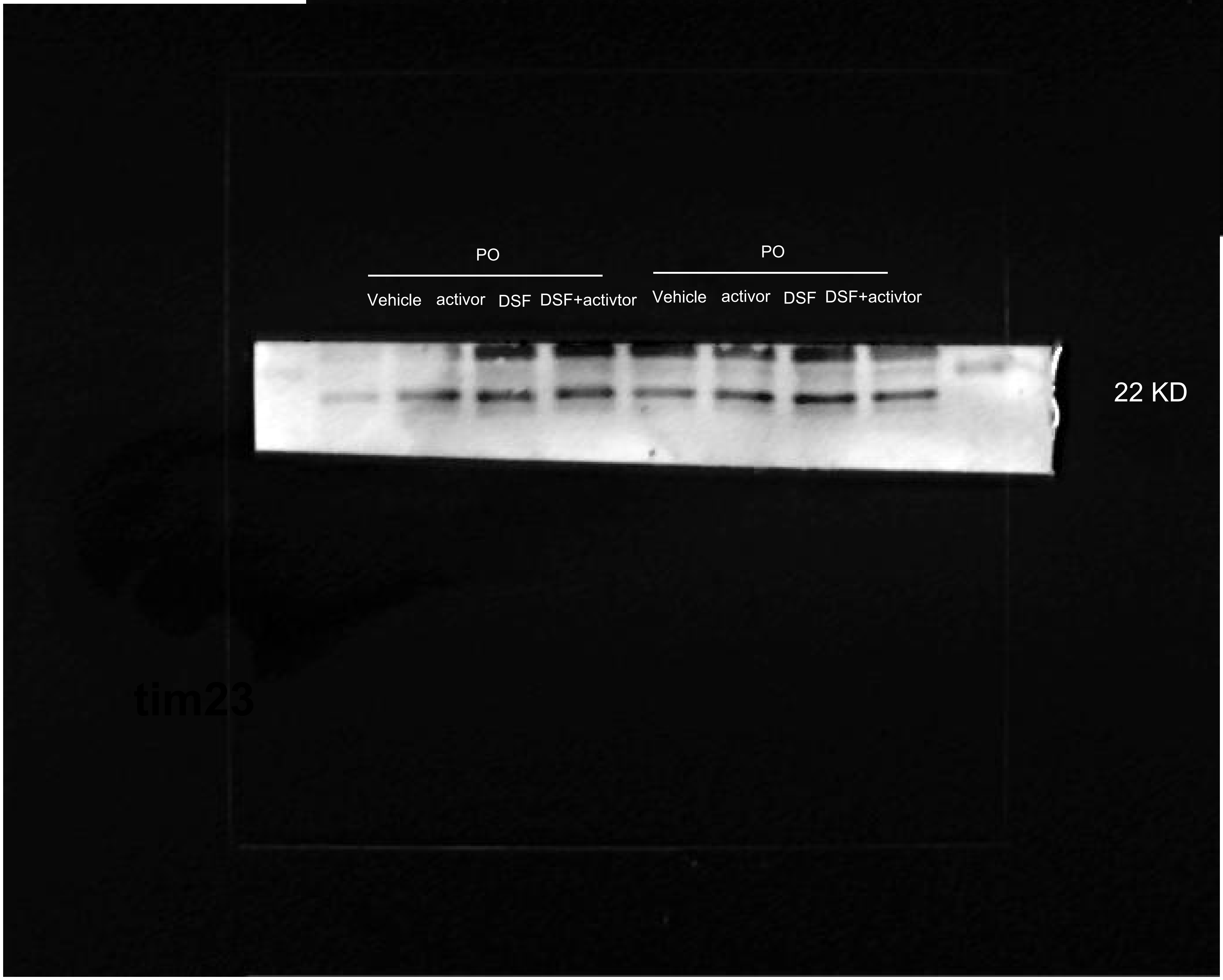

cell P62 Fig 7D

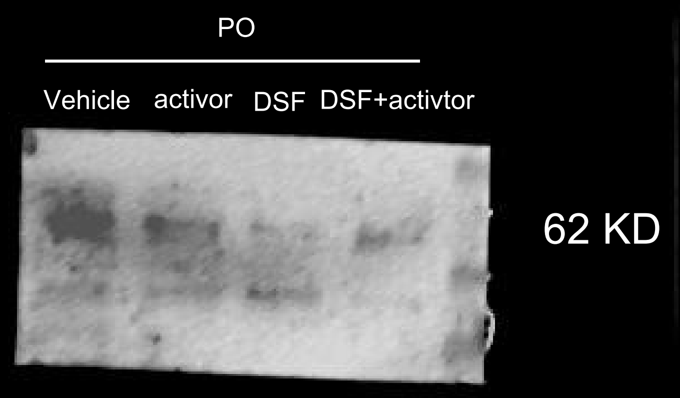

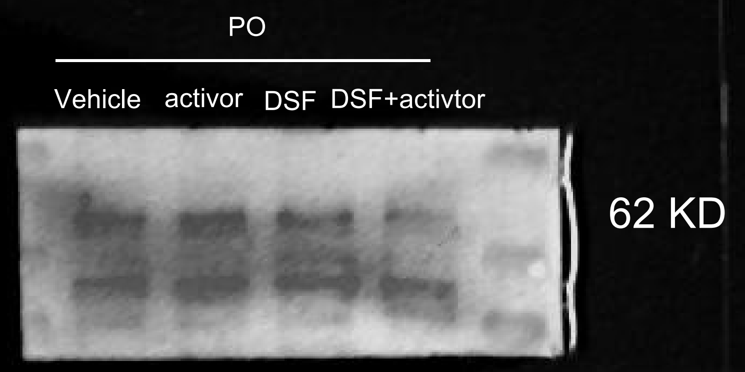

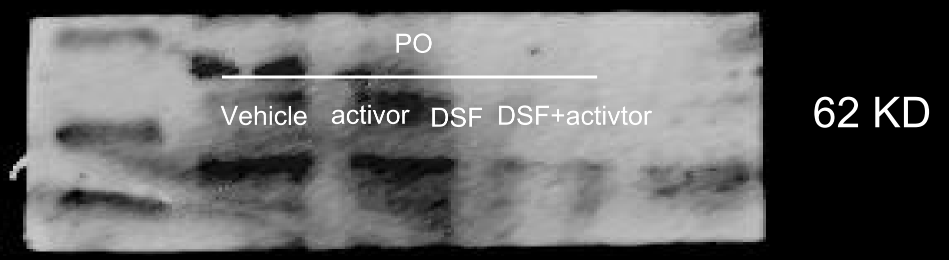

cell TOMM40 Fig 7D

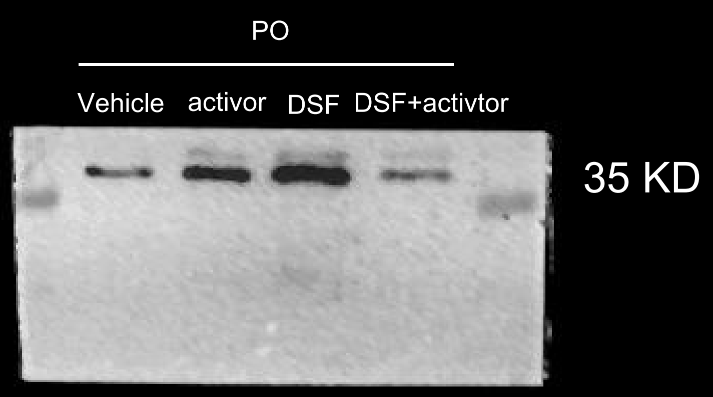

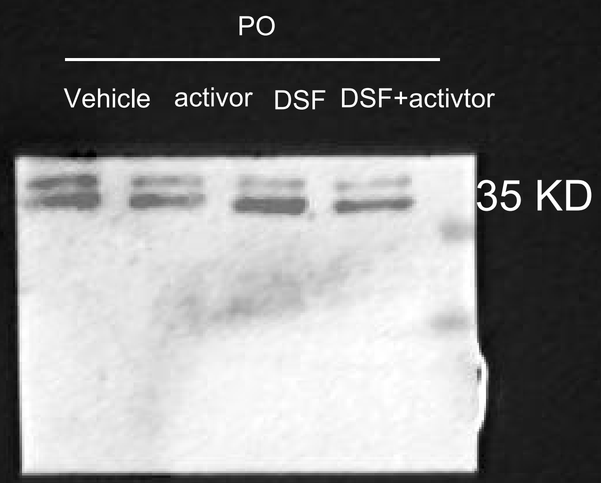

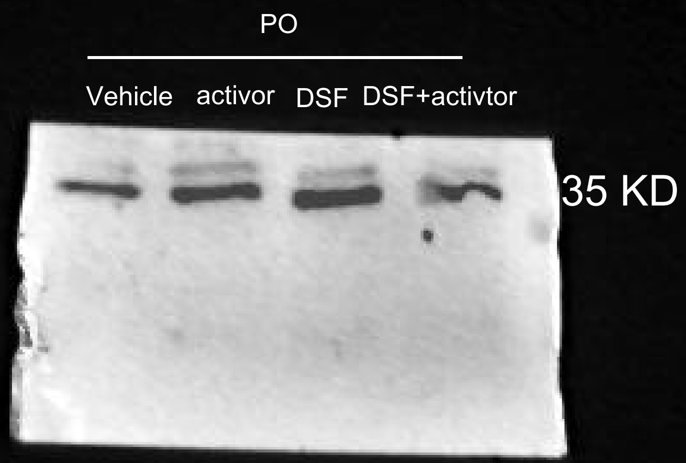

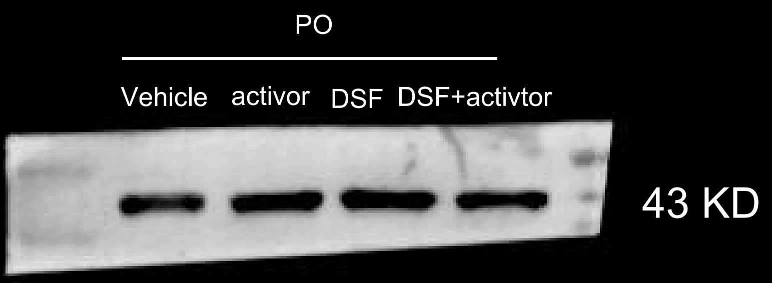

cell β-actin Fig 7D

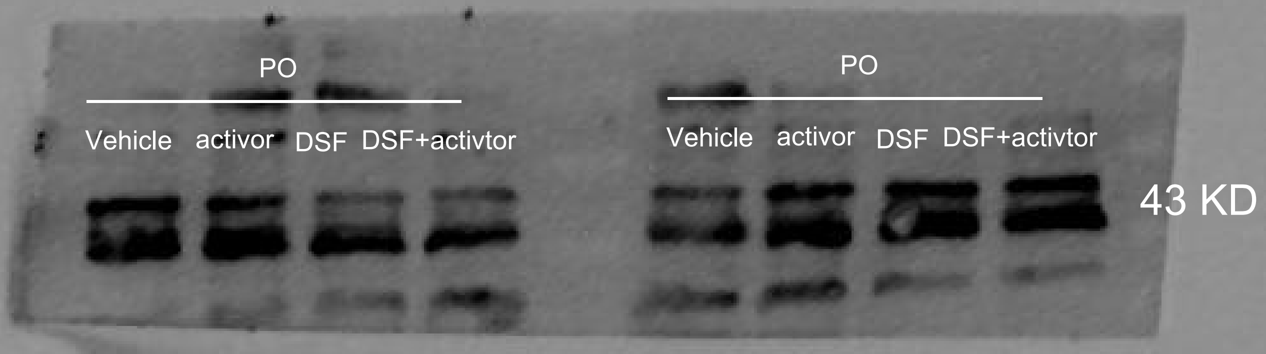

Supplement: Supplementary file 1 [file antioxidants-15-00867-s001.zip › antioxidants-4365249-supplementary.pdf]
